# CORTADO: hill climbing optimization for cell-type specific marker gene discovery and clustering accuracy improvement

**DOI:** 10.1093/bioadv/vbag106

**Published:** 2026-04-13

**Authors:** Musaddiq K Lodi, Leiliani Clark, Satyaki Roy, Preetam Ghosh

**Affiliations:** Virginia Commonwealth University Integrative Life Sciences, Richmond, VA, 23220, United States; Virginia Commonwealth University, Center for Biological Data Science, Richmond, VA, 23220, United States; University of Alabama in Huntsville, Department of Mathematical Sciences, Huntsville, Alabama, 35899, United States; Virginia Commonwealth University, Department of Computer Science, Richmond, VA, 23220, United States

## Abstract

**Motivation:**

The advent of single-cell RNA sequencing (scRNA-seq) has enhanced our ability to study cellular heterogeneity. Accurately identifying distinct subpopulations and their defining markers is critical for understanding tissue diversity. We introduce CORTADO, a hill-climbing optimization framework for marker discovery and clustering refinement.

**Results:**

CORTADO maximizes differential expression, minimizes redundancy via cosine similarity, and enforces sparsity for interpretability. By using CORTADO-selected markers to inform the cell-type identification process, an iterative refinement approach markedly increases the Adjusted Rand Index (ARI), a metric that quantifies how well the clustering assignments align with gold-standard cell-type annotations. Benchmarking across brain, immune, spatial, and cancer datasets confirms that CORTADO delivers biologically relevant markers and consistently outperforms state-of-the-art methods in both marker discovery and clustering accuracy.

## Introduction

The emergence of single-cell RNA sequencing (scRNA-seq) has enabled researchers to explore transcriptional processes at single-cell resolution. This technology has been instrumental in identifying rare cell types, evaluating cellular heterogeneity, and measuring variations between individual cells (Wu *et al*. 2018). Single-cell biology focuses on characterizing cellular heterogeneity by uncovering distinct sub-populations (Zhang *et al*. 2023). Several methods have been developed to perform this task, ranging from unsupervised clustering to reference dataset-based cell annotation (Yu *et al*. 2022, Lodi *et al*. 2024a, 2024b). A crucial aspect of delineating cell types involves identifying marker genes, whose expression is significantly enriched in specific cell populations relative to others. These genes serve as molecular signatures, enabling the classification of cellular identities and functions. Such *marker genes* not only facilitate the distinction between closely related cell types but also provide insights into lineage relationships, developmental trajectories, and functional specialization.

Marker genes, while variably defined in the literature without a universal consensus, serve the essential purpose of distinguishing between different cell populations. A recent benchmarking work of marker gene selection methods notably defined marker genes as a subset of the differentially expressed genes of a cluster that have the most relevance to the cell type’s biological function (Pullin and McCarthy 2024). The most common tool for marker gene identification is log fold change-based statistical testing, such as the Wilcoxon Rank Sum and *t*-Test (Wolf *et al*. 2018), and it is widely used in scRNA-seq workflows (Wolf *et al*. 2018, Butler *et al*. 2018).

Calculating the differential expression of a gene alone may not reliably identify a set of marker genes for a specific cluster. For instance, a gene might show high expression in the target cell type but also moderately high expression in another cell type, with low expression in the rest. Existing approaches leveraging expression-based statistical methods might still identify this gene as a marker, even though it is not exclusive to the target cell type, highlighting the need for a more thorough assessment of gene expression patterns for nuanced marker gene selection. Several marker gene selection methods have emerged in recent times. One method, RankCorr, identifies marker genes by calculating the Spearman correlation with the cluster indicator vector for each fixed cluster and using that information to identify appropriate markers (Vargo and Gilbert 2020). Another technique, scMAGS, utilizes differential expression or variability to calculate cluster-specific marker scores and cluster validity indices, the Silhouette index or Calinski-Harabasz index to select such marker genes (Baran and Doğan 2023). COSG introduced the importance of considering the cosine similarity profile of genes in marker selection (Dai *et al.* 2022).

More recent methods have been developed to address the marker gene problem as well, since publication of the comprehensive Pullin and McCarthy benchmarking study (Pullin and McCarthy 2024). One such method, CosGeneGate, focuses on label-free marker gene selection, which ignores cell-cluster labels and instead discovers genes that best distinguish cell-populations (Liu *et al*. 2024). Another method, a hierarchical clustering framework for marker gene identification, focuses on inherent relationships between cell-types to derive marker genes. By leveraging information that relates cell-types by lineage, this method is able to detect genes that group related cell-types by their shared markers (Sun and Qiu 2024). An exciting direction of marker gene selection is capturing non-linear relationships in genes. MarkerMap leverages a supervised learning strategy to inform learned marker genes that are consistent with related cell and tissue types (Gregory *et al*. 2024). Together, these recent methods demonstrate the growing emphasis on incorporating biological structure and machine learning into marker gene discovery. However, inevitable trade-offs between interpretability, scalability, and consistency across datasets remain evident, highlighting the need for approaches that can robustly identify biologically meaningful and discriminative markers across heterogeneous single-cell datasets.

Current approaches to marker gene selection often fail to provide a holistic view of differential gene expression while adequately addressing overlap or redundancy in gene expression profiles (Chaitankar *et al*. 2010a, 2010b, Nalluri *et al*. 2017). Additionally, existing methods typically rely on ranking genes by *P*-adjusted values, failing to identify a concise and biologically meaningful set of marker genes. To address the lack of conciseness and specificity in gene selection, we present a hill Climbing OptimizaTion foR cell-type specific mArker gene DiscOvery and Clustering Accuracy Improvement, *CORTADO*, a framework that selects markers by optimizing three properties: differential expression in the clusters of interest, distinctiveness in their expression profiles (or non-redundancy) concerning other markers, and sparseness, that is, minimizing the number of selected genes. By actively discouraging the selection of genes with highly similar co-expression patterns, CORTADO minimizes overlap in expression profiles and ensures that selected markers exhibit unique expression signatures, capturing diverse functional roles within the cellular landscape.

In addition to marker selection, CORTADO introduces an *iterative refinement strategy* that leverages selected markers to directly improve clustering assignments. In this approach, CORTADO markers are used as a reduced feature set for community detection, and the process is repeated until convergence. Crucially, this dual design allows CORTADO to function both as a marker discovery and clustering enhancement tool, linking the quality of selected markers directly to improvements in cell-type resolution.

CORTADO also introduces flexibility that most existing methods lack, supporting both *constrained* and *unconstrained* scenarios for marker selection. In the constrained scenario, users can specify the number of marker genes to be identified, enabling tighter control over the size of the gene set. This flexibility makes CORTADO adaptable to diverse analytical needs and scalable to datasets with varying complexity. Moreover, the hill-climbing optimization framework underpinning CORTADO efficiently balances the need for non-redundant and sparse marker sets, addressing redundancy in gene expression profiles without compromising the biological relevance of the selected markers.

Through thorough enrichment analyses, clustering benchmarks, and the examination of spatial localization-based expression, we establish the biological relevance and uniqueness of CORTADO-selected markers across diverse datasets. On the DLPFC 151507 dataset (Pardo *et al*. 2022), derived from spatial transcriptomics data of the dorsolateral prefrontal cortex, CORTADO identifies GFAP, a well-known marker of astrocytes. In the Zeisel mouse brain dataset (Zeisel *et al*. 2015), containing 3005 cells from hippocampal tissue, CORTADO highlights unique genes associated with the “Modulation of the Chemical Synaptic Transmission” pathway, underscoring its capacity for non-redundant selection in neuronal communication pathways. Analysis of a peripheral blood mononuclear cell dataset reveals that the selected markers are enriched in pathways such as viral transcription and immune response, showcasing CORTADO’s ability to identify significant genes with clinical relevance (Hoffman 2024). CORTADO is implemented in Python and available for installation at https://www.github.com/lodimk2/cortado-marker.

In this study, we present the following contributions:

- We introduce CORTADO, a novel marker gene selection method that optimizes differential expression while minimizing redundancy through cosine similarity, ensuring a biologically meaningful and non-redundant marker set.
- Existing marker gene selection methods typically emphasize either differential expression or redundancy reduction, often leading to inconsistent performance across datasets with varying characteristics. CORTADO is the first to integrate both criteria to achieve robustness across diverse datasets and evaluation metrics.
- We extend CORTADO with an iterative refinement procedure that improves clustering accuracy by repeatedly using selected markers as features for community detection, showing its dual role in marker discovery and clustering improvement.
- We validate CORTADO’s utility by identifying clinically relevant marker genes in a skin cancer dataset, highlighting its use for biological and clinical discovery.

## Results

### CORTADO overview

**Figure 1:**
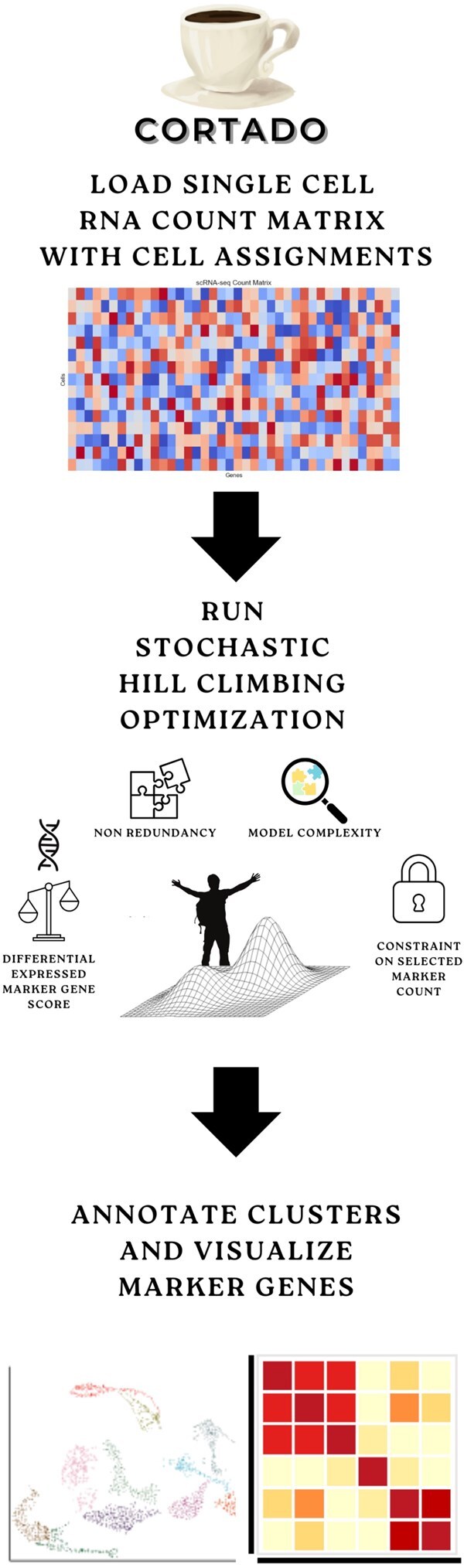
Overview of the CORTADO framework for cell-type-specific marker gene discovery. The CORTADO pipeline begins with a single-cell RNA sequencing (scRNA-seq) count matrix and corresponding cell-type or cluster assignments. It then applies a stochastic hill-climbing optimization algorithm to identify an optimal set of marker genes by jointly maximizing differential expression, minimizing redundancy across clusters, and controlling for marker set complexity. The optimization is guided by constraints on marker count, a distinctiveness objective, and overall model simplicity. The resulting marker genes can be used to annotate clusters and visualize expression patterns across the dataset, enabling interpretable and biologically meaningful insights into cellular heterogeneity.

CORTADO, implemented using *stochastic hill-climbing optimization*, was evaluated for its ability to select cluster-specific marker genes across diverse scRNA-seq datasets and to enhance clustering accuracy through iterative refinement. The raw count matrix and a metadata file containing clustering assignments underwent processing to create an AnnData object (Wolf *et al*. 2018). Marker genes and their cosine similarity scores were calculated for each user-specified cluster of interest, with additional functionality available for identifying markers across clusters. As depicted in Fig. 1, CORTADO operates under an objective function comprising three weighted terms (see “Methods” section for details). The first term, $c_{1}$, prioritizes genes with strong differential expression in the cluster of interest compared to other clusters. The second term, $c_{2}$, penalizes the selection of gene pairs with high cosine similarity, thereby promoting diversity among the chosen markers. The third term, $c_{3}$, discourages the selection of an excessive number of genes, enforcing sparsity in the final marker set. Each term is weighted by a user-defined parameter $\lambda_{i}$ (i=1,2,3), with larger λ values assigning greater importance to the corresponding term, making the overall objective function: $\lambda_{1}c_{1}-\lambda_{2}c_{2}-\lambda_{3}c_{3}$.

Based on a sensitivity analysis using simulated datasets with both well-separated and overlapping clusters, we recommend parameter values of $\lambda_{1}=0.9$ and $\lambda_{2}=0.1$ for most scRNA-seq datasets. Details of this analysis are provided in the Supplementary Materials, Section 1. To determine the effect of $\lambda_{3}$ on the number of genes selected when the model is unconstrained, we show that increasing the value of $\lambda_{3}$ decreases the number of genes chosen across clusters in the Zeisel Mouse Brain dataset (Zeisel *et al*. 2015). To benchmark CORTADO, we evaluated both its marker discovery and iterative refinement performance across multiple scRNA-seq datasets, including the Zeisel Mouse Brain dataset, the Baron Pancreas dataset, the Muraro Pancreas dataset, and the PBMC3K dataset (Zeisel *et al*. 2015, Baron *et al*. 2016, Muraro *et al*. 2016, Hoffman 2024). We also applied CORTADO to a Basal Cell Carcinoma (BCC) dataset (Yost *et al*. 2019) to assess its utility in identifying clinically relevant markers.

### CORTADO outperforms baseline methods in marker gene selection and clustering accuracy

#### Key considerations

To evaluate the performance of CORTADO, we benchmarked it against existing marker gene selection methods using both quantitative metrics and downstream clustering accuracy. We first devised two scores to assess marker gene strength: the *log ratio difference* and the *median cosine similarity*. The log ratio difference is used as a marker gene score, measuring the expression difference of selected marker genes in the cluster of interest versus all other clusters, while cosine similarity evaluates the similarity of genes within the cluster of interest relative to other clusters (see “Methods” section for details).

While Pullin and McCarthy (2024) proposed log ratio difference as a key indicator based on the assumption that strong markers have high expression differences in the target cluster, simple differential expression may not fully capture marker gene quality, as markers should distinguish cell subpopulations beyond differential expression alone. To address this, CORTADO leverages cosine similarity, a scale-free measure, to identify genes with diverse profiles outside the target cluster (Dai *et al.*  2022). Importantly, cosine “dissimilarity” provides a measure of the lack of overlap in expression profiles, improving distinction across subpopulations.

Another key aspect for evaluating a marker gene selection method is determining how many unique genes compared to other methods, a method can select. Selecting novel marker genes is a key aspect of biomarker discovery, as methods that identify a higher proportion of exclusive markers demonstrate a more distinctive selection strategy and greater potential for uncovering previously unreported cell-type signatures. Methods that converge heavily on shared genes, while potentially robust, may offer limited added value in discovery-oriented settings where identifying novel candidates is the primary goal. However, it is still crucial that the unique marker genes selected by a method perform well on standard benchmarking metrics. Hence, we opted to compare method performance by evaluating both all genes selected by a method, and the unique genes selected by a method compared to all other methods.

CORTADO was compared to four baselines across seven datasets using these metrics. To ensure a fair comparison against all methods, we selected the top 5 marker genes for each method. For COSG and RankCorr, we selected the top 5 genes sorted directly from their method (Dai *et al.*  2022, Vargo and Gilbert 2020). For the Scanpy DE methods, we first applied a cutoff on the FDR value of *P* < .05. Then, we sorted by descending log2 fold change and selected the top 5 genes. To select unique genes per method, this was aggregated across clusters, and genes were only kept if they were not selected by any other method.

#### Unique marker selection

**Figure 2:**
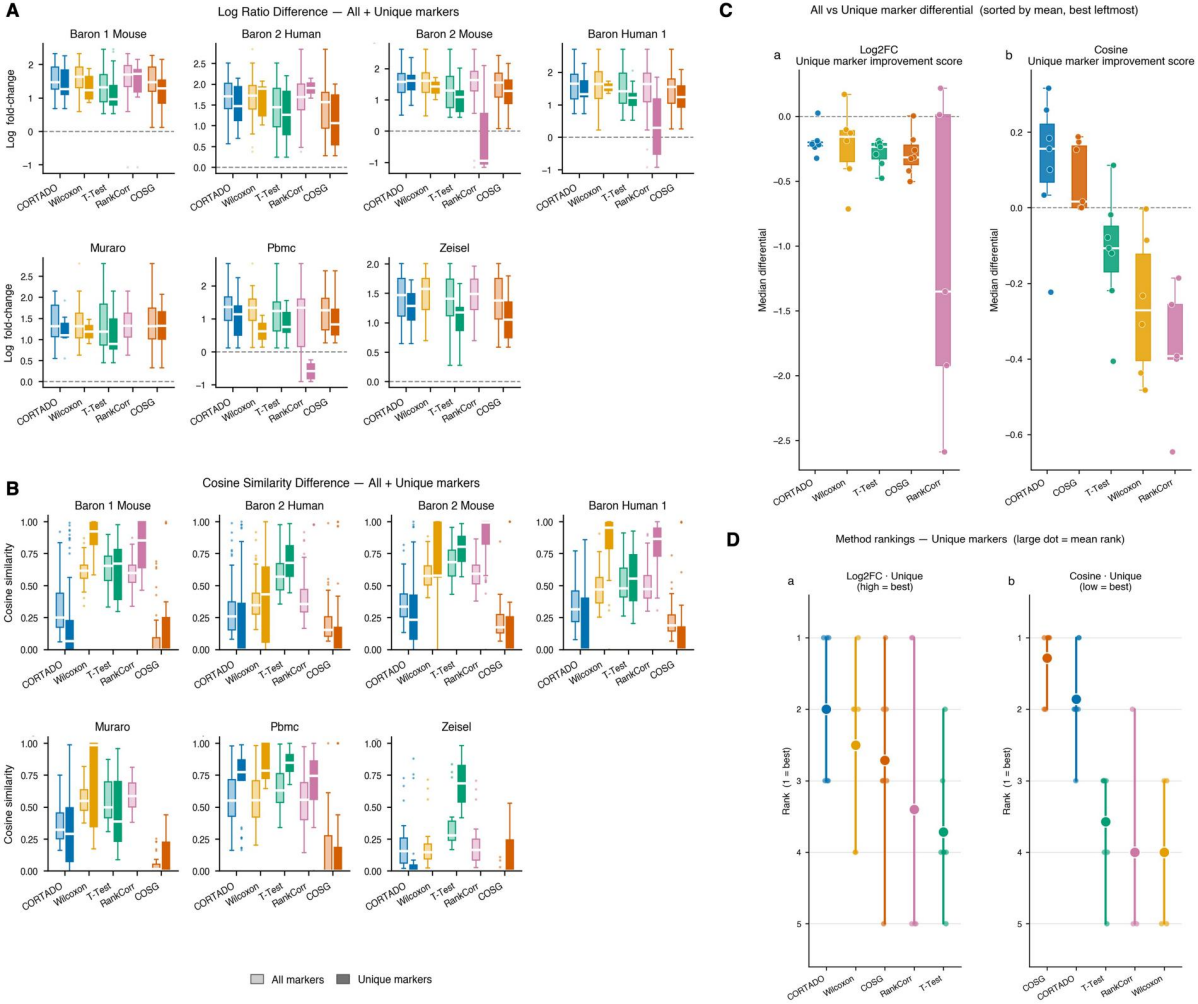
Performance comparison across 7 datasets based on log-ratio difference score and cosine similarity score, considering all selected markers by each method and unique markers. We selected 5 markers per cluster per method. (A) Comparison of performance for each method on the 7 benchmarking datasets for the log ratio difference score; solid color boxes indicate performance only on unique markers, while the transparent color indicates performance on all markers selected by the method. A higher score on this metric indicates better performance. (B) Comparison of performance for each method on the 7 benchmarking datasets for the cosine similarity difference score; solid color boxes indicate performance only on unique markers, while the transparent color indicates performance on all markers selected by the method. A lower score on this metric indicates better performance. (C) Comparison of marker differential score for each method for both log ratio difference and cosine similarity difference scores. This metric is calculated for each dataset, the difference in medians between all marker performance and unique marker performance. A differential score greater than 0 indicates the method performs better on its unique marker set. (D) Performance ranking on unique marker sets across all datasets for each method. The large dot on the line indicates the method’s mean ranking with respect to other methods.

From the results in Fig. 2A, we see that all methods perform reasonably similarly when considering all selected genes on the log ratio difference score, demonstrating their shared ability to select marker genes that are highly expressed in the cluster of interest and lowly expressed in others. In contrast, when considering only the unique marker genes selected by each method, a much clearer performance gap emerges; CORTADO is the best-performing method on four of the seven benchmarking datasets, more than any other method. Notably, RankCorr was unable to select unique markers for the Muraro Pancreas and Zeisel Mouse Brain datasets, and experienced a substantial performance drop on three additional datasets, suggesting that when it does select unique marker genes, these markers may not be biologically informative (Zeisel *et al*. 2015, Muraro *et al*. 2016, Vargo and Gilbert 2020). When considering the cosine similarity difference score (Fig. 2B), a clearer performance separation is observed for both all and unique markers; CORTADO and COSG consistently outperform all other methods, and notably exhibit less variation between their performance on all markers versus their unique marker subsets, suggesting their exclusive gene selections remain geometrically discriminative in expression space.

Figure 2C shows the unique marker improvement score for each method across all datasets, defined as the difference in median performance between a method’s full marker set and its unique marker subset. A score above zero indicates that a method’s exclusive markers outperform its full set, while a score below zero indicates the shared markers are driving performance. For the log ratio difference score, all methods score below zero, confirming that shared markers contribute meaningfully to specificity across all methods. However, we note that CORTADO has much less variation and more consistent performance compared to all other methods. RankCorr is a clear outlier, with a strongly negative score indicating its unique markers are substantially less informative than those it shares with competing methods. or the cosine similarity score, CORTADO is the strongest performer, achieving the highest improvement score across datasets, meaning its exclusive markers produce better cluster separation than its full marker set. COSG also achieves a modest positive score, while all remaining methods score below zero. This suggests that these methods select exclusive genes that are genuinely discriminative rather than redundant.

To empirically rank performance, we took the median of the metrics across clusters per dataset. A method that performed the best on a metric for a given dataset is given a rank of one, and so on. A higher median log ratio and a lower median cosine similarity correspond to a better rank (with 1 being the best). We based the ranking exclusively on unique marker performance to eliminate inter-method redundancy and enable a direct comparison of method-specific gene selection. As shown in Fig. 2D, CORTADO achieves the top rank for log ratio difference with minimal variability, outperforming other methods, while consistently ranking second in cosine similarity difference. Unsurprisingly, COSG performs best in cosine similarity but ranks in the middle on log ratio difference, highlighting the trade-offs of existing approaches. Overall, CORTADO excels at identifying genes that are both strongly differentially expressed and diverse in their expression profiles.

#### Clustering accuracy

Beyond marker selection, we assessed whether these markers could also enhance clustering accuracy through an *iterative refinement strategy*, when compared to ground-truth labels. In this procedure (detailed in Algorithm 1), we first initialize clustering assignments using Scanpy with default parameters (see “Scanpy DE methods: Wilcoxon Rank Sum and *t*-test” section). For each cluster, we then select 10 CORTADO markers, pool them together, and re-cluster the dataset using only these markers. This process is repeated for a user-defined maximum number of iterations, or until convergence, defined as an Adjusted Rand Index (ARI) of at least 0.95 when compared to the previous iteration (Hubert and Arabie 1985). ARI measures how consistently pairs of cells are assigned to the same or different clusters in two partitions. An ARI of 1 indicates identical clustering. An ARI of 0.95 indicates that only about 5% of pairwise relationships differ between the two clusters. ARI, while being the field accepted metric for evaluating single cell clustering performance, is susceptible to misleading results if there is a class imbalance in the dataset. Thus, we chose to evaluate on datasets that have a more even distribution of cell-types, are moderately sized, and do not contain any rare cell-types. This directly links marker quality to improved clustering resolution, showing the dual functionality of CORTADO as both a marker discovery and clustering enhancement framework.

**Figure 3:**
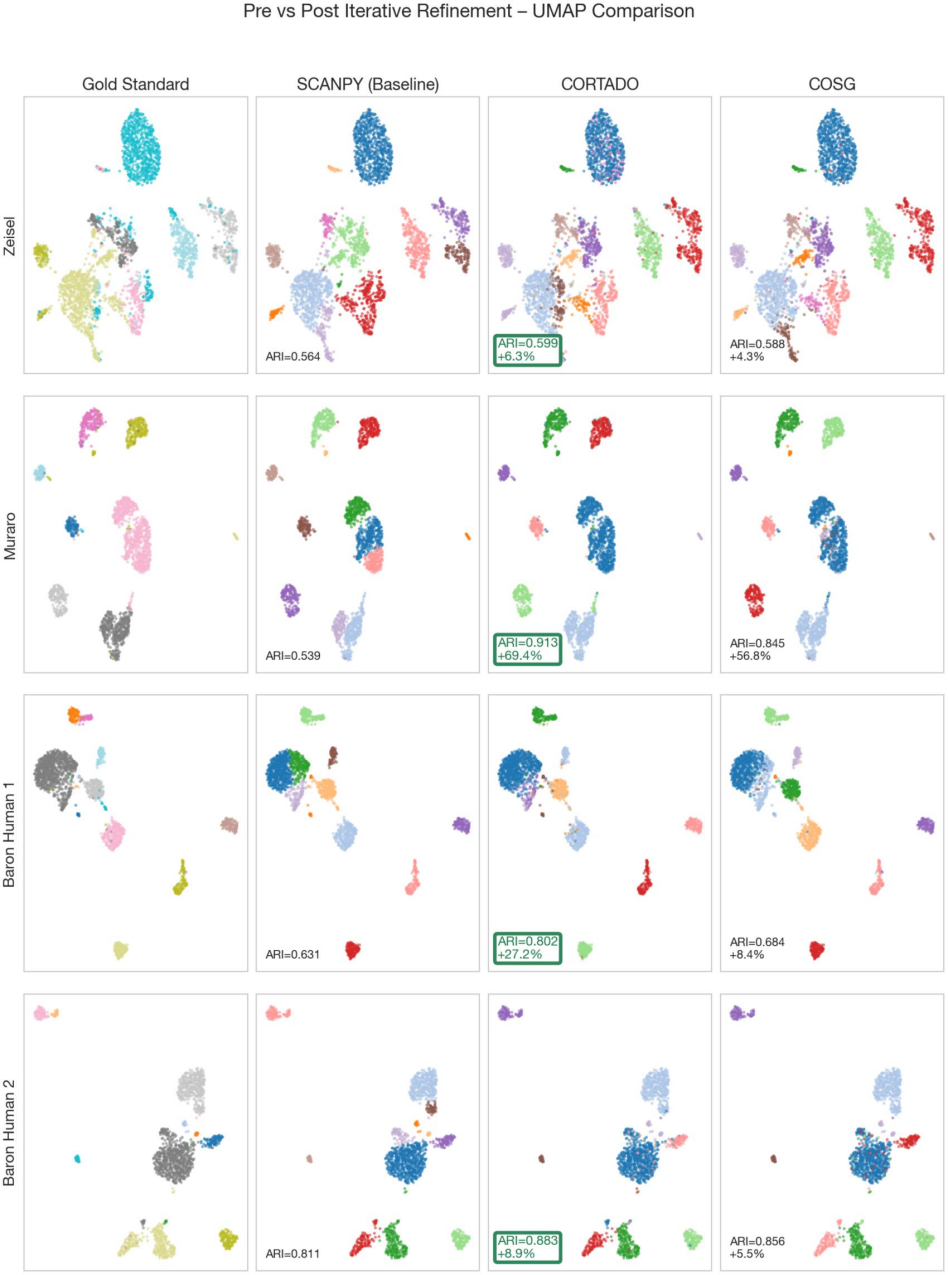
Percentage improvement of Adjusted Rand Index (ARI) for CORTADO and COSG compared to standard Scanpy clustering.

To benchmark this iterative refinement, we compared CORTADO-selected markers against COSG-selected markers, since both methods performed strongly in the quantitative benchmarking. We evaluated clustering improvements on four scRNA-seq datasets: Baron Human 1, Baron Human 2, Zeisel Mouse Brain, and Muraro Pancreas, all of which include ground-truth annotations for gold-standard evaluation. We compared the clustering assignments for the baseline SCANPY clustering and the post-iterative refinement methods (CORTADO and COSG marker clustering) to the ground-truth clusters provided by the authors of the original datasets. Figure 3 shows the percent improvement in ARI relative to standard Leiden clustering.

By using CORTADO-selected marker genes as the feature space for Scanpy clustering, we show that CORTADO delivers consistent and substantial improvements in clustering accuracy across all benchmarking datasets, outperforming COSG in every comparison. Strikingly, clustering results generated from only CORTADO markers not only match but surpass the performance of standard Leiden clustering applied to thousands of highly variable genes, demonstrating that a small, carefully optimized marker set can outperform conventional high-dimensional approaches. This result highlights a key advance: CORTADO-selected markers are directly responsible for enhancing cell-type separability, contributing to a central goal of single-cell analysis. In other words, the markers selected by CORTADO are more discriminative than markers selected by that of COSG, making them more valuable for downstream classification of cell-types.

### CORTADO identifies spatially localized marker genes with biological relevance

We assess the ability of CORTADO to select genes in a spatial dataset context by analyzing the DLPFC 151507 dataset, which is a spatial transcriptomics dataset derived from the dorsolateral prefrontal cortex (DLPFC), a critical brain region involved in executive functions and working memory. Generated using the 10× Genomics Visium platform, this dataset provides spatially resolved gene expression profiles for approximately 3639 spots, each linked to specific spatial coordinates within the tissue. It includes information on six cortical layers annotated through histological analysis, enabling the exploration of cellular heterogeneity and tissue organization (Pardo *et al*. 2022).

**Figure 4:**
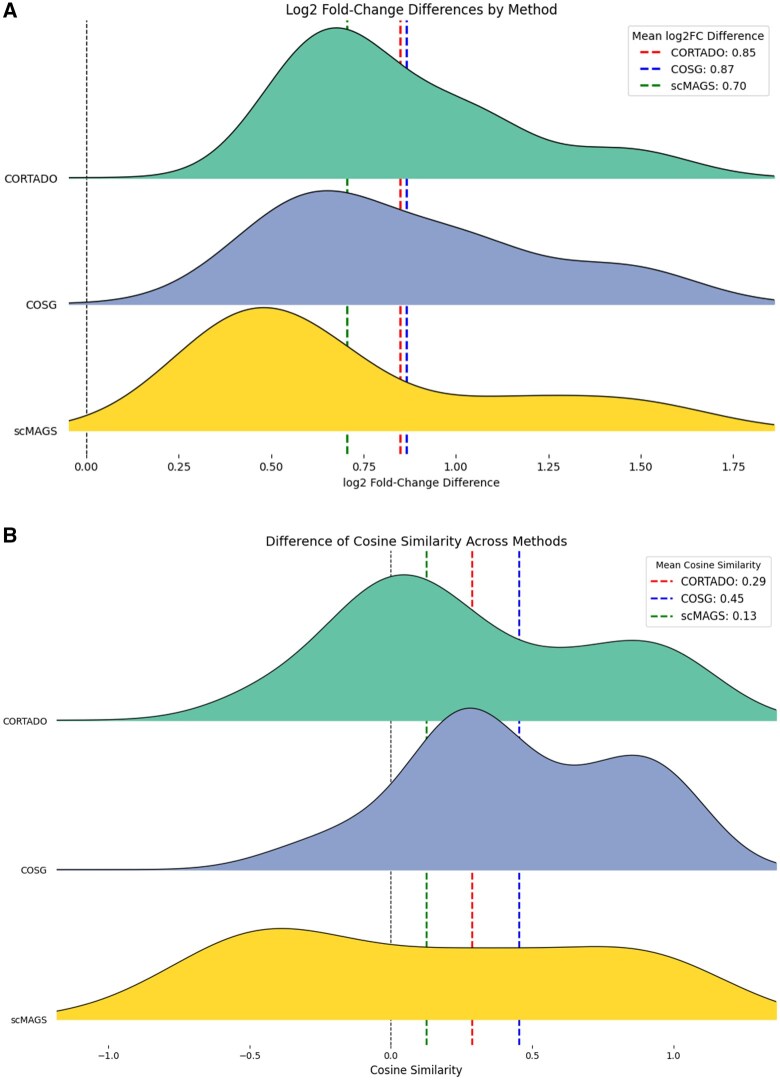
CORTADO analysis on the DLPFC 151507 Dataset. (A) Log Ratio difference score density plot for markers selected by all three methods. The dotted line corresponds to the mean log ratio difference. (B) Cosine similarity between the mean expression of markers selected by the method for each cluster compared to other clusters, visualized as a density plot. The dotted line corresponds to the mean cosine similarity score.

This experiment aims to show that the expression of markers selected by CORTADO, compared to other methods, was more spatially localized, with high expression in the location of interest and low expression in the others. For fairness, we compared against two methods specifically optimized for performance on spatial transcriptomic datasets, scMAGS and COSG (Baran and Doğan 2023, Dai *et al.* 2022). To quantitatively assess the performance of CORTADO compared to the baseline methods, we used the log ratio difference score and cosine similarity scores defined in the “Methods” section. In Fig. 4A, we see that CORTADO and COSG have the best performance in terms of the log ratio score, meaning that both methods are choosing well-separated genes based strictly on gene expression differential. scMAGS lags notably behind in this experiment. When analyzing based on the cosine similarity score (Fig. 4B), where a lower value indicates stronger performance, scMAGS is the best-performing method, with CORTADO in second and COSG a distant third. COSG, while having a strong log ratio difference performance, selects genes that are very similar in expression to markers in other clusters, showcasing a lack of unique selection in this method. We can conclude from the quantitative benchmarking on this spatial dataset that CORTADO performs strongly across both evaluation metrics (Dai *et al.*  2022, Baran and Doğan 2023).

**Figure 5:**
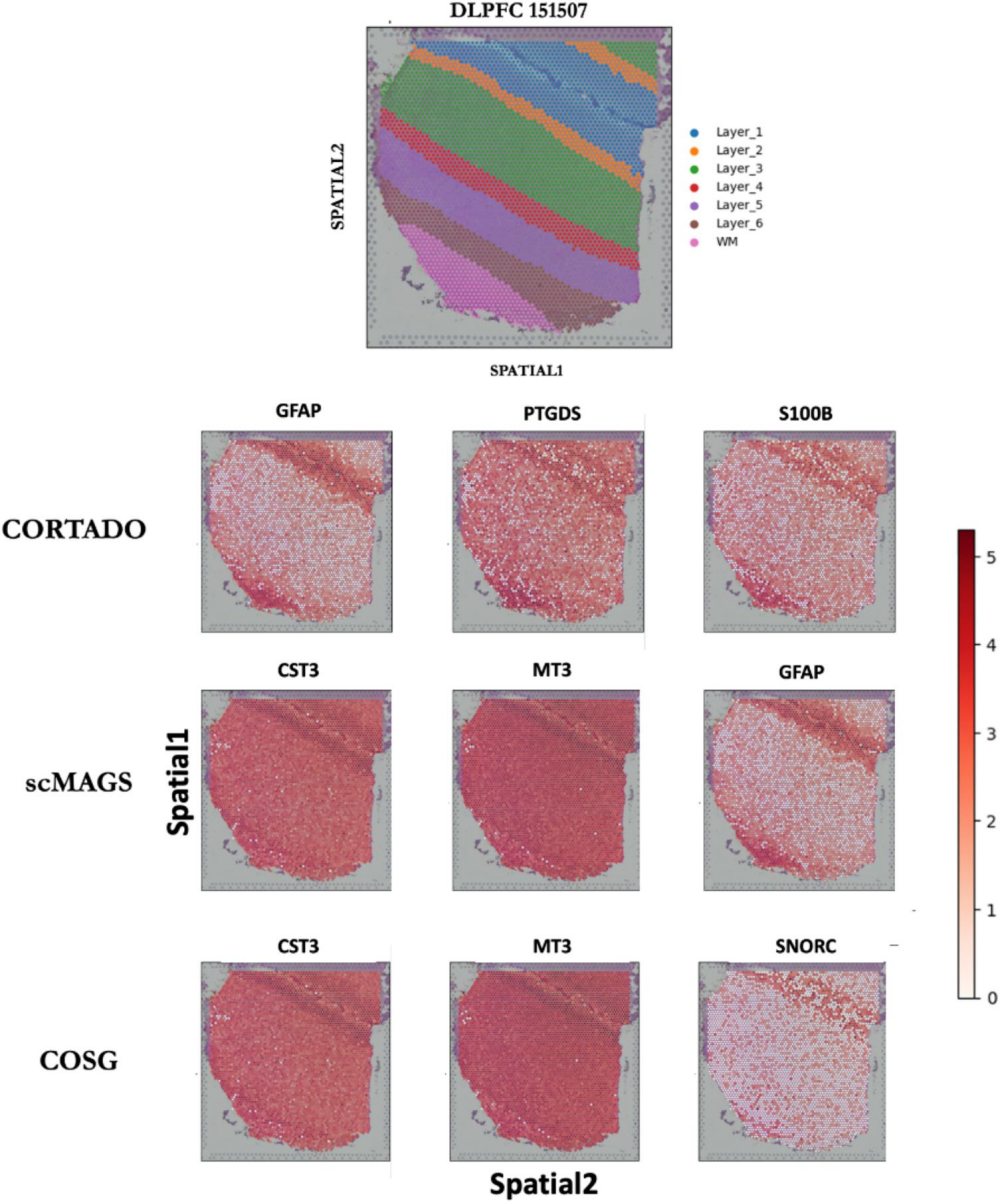
Expression localization of selected markers for CORTADO, scMAGS, and COSG on Layer 1 of DLPFC 151507 spatial dataset.

When examining the spatial localization-based expression, we looked at the top three markers selected by each method for an example layer, Layer 1. We visualize the localization of expression of these markers in Fig. 5. It is clear from this experiment that the markers selected by CORTADO are heavily localized to Layer 1, with the majority of their expression being concentrated there. GFAP and S100B, in particular, are very specifically isolated to Layer 1. The markers selected by CORTADO, compared to the scMAGS and COSG, have biological significance. CORTADO selected GFAP as its most significant marker; GFAP is a well-studied marker of prefrontal cortex cells (Si *et al*. 2004). Abnormal expression of GFAP in the prefrontal cortex can lead to psychotic illnesses such as schizophrenia and bipolar disorder (Feresten *et al*. 2013). S100B, which was only selected by CORTADO, is a canonical marker of DLPFC cells categorized by numerous studies. The upregulation of S100B is used to separate DLPFC cells from other cells in the brain, particularly in scRNAseq studies (Zhai *et al*. 2012, Zhang *et al*. 2021, Michetti *et al*. 2023). scMAGS and COSG, on the other hand, selected CST3 and MT3, which have high expression across all Layers, and not just Layer 1 (Baran and Doğan 2023, Dai *et al.*  2022). This makes the markers selected by the scMAGS and COSG inherently poor at distinguishing Layer 1 from other clusters. These results demonstrate CORTADO’s superior performance on spatial transcriptomics datasets.

### CORTADO finds novel biomarkers in basal cell carcinoma

**Figure 6:**
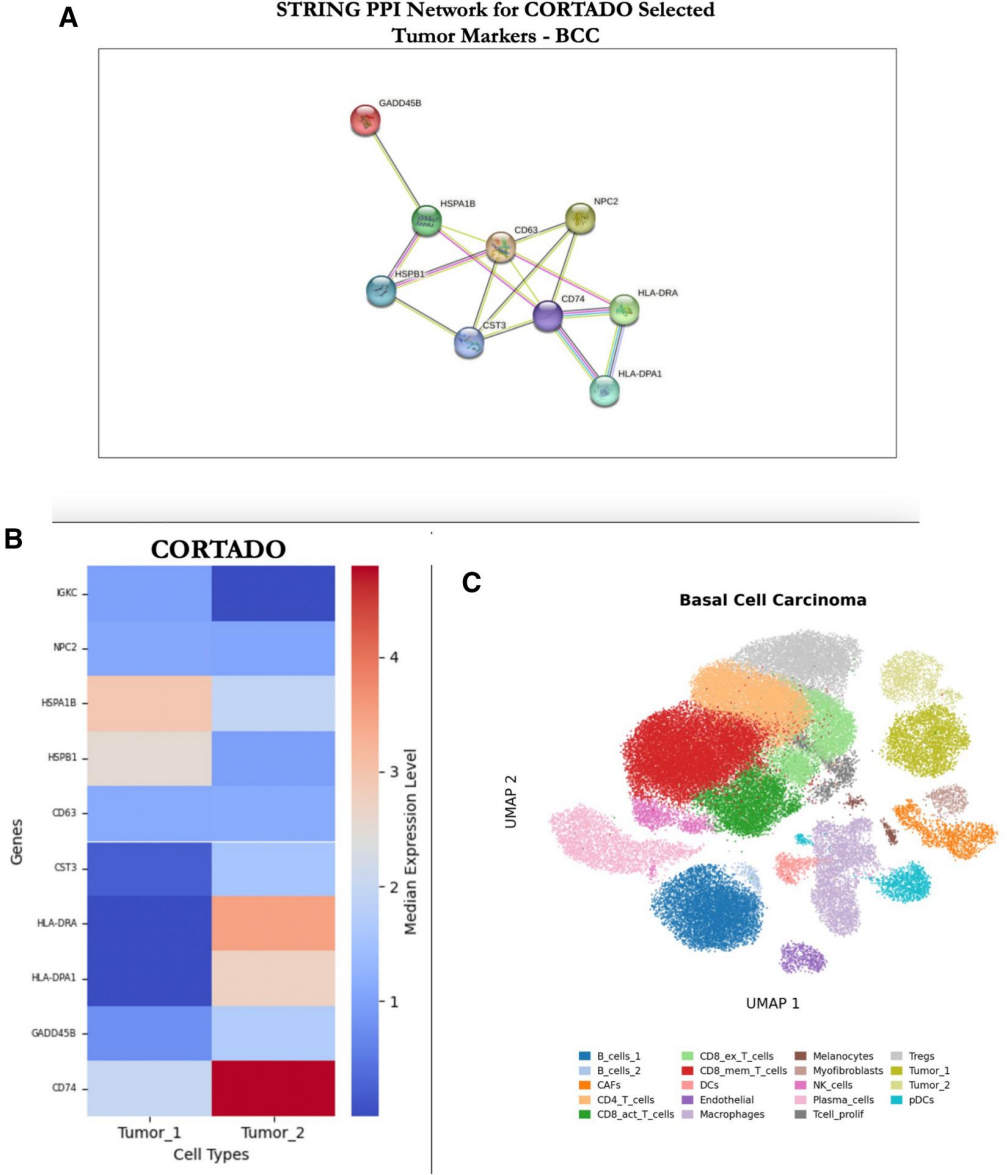
CORTADO Analysis on Basal Cell Carcinoma (BCC) dataset. (A) STRING database Protein-Protein Interaction (PPI) network analysis on CORTADO selected markers for Tumor 1 and Tumor 2 clusters. (B) Median Expression for CORTADO selected markers in Tumor 1 and Tumor 2 clusters. (C) UMAP Visualization for the BCC dataset, which contains 19 clusters. We focused the CORTADO Analysis on the Tumor 1 and Tumor 2 clusters.

The basal cell carcinoma (BCC) dataset contains 53 030 cells from biopsies collected from primary tumor sites of 11 patients with histologically proven advanced or metastatic BCC (Yost *et al*. 2019). This dataset comprises 19 distinct cell clusters, including 2 malignant clusters, and 577 identified genes (Yost *et al*. 2019). To investigate the T cell response to checkpoint blockade immunotherapy (anti-PD-1 therapy) in cancer patients, paired single-cell RNA (scRNA) and T cell receptor (TCR) sequencing were performed (Yost *et al*. 2019). The findings suggest that the efficacy of checkpoint blockade immunotherapy may rely more on newly recruited T cells than on the reactivation of pre-existing T cells within the tumor (Yost *et al*. 2019). CORTADO was applied to the Tumor 1 and Tumor 2 malignant clusters, and the to selected markers for each cluster were analyzed for relevance to skin cancer. The full list of clusters in the dataset is shown in Fig. 6C.

The top selected markers from the Tumor 1 cluster, shown in Fig. 6B, affect the survival of melanoma cells contingent on their expression levels. HSPA1B, a prognostic biomarker in skin cutaneous melanoma, influences the regulation of the immune response to melanoma cancer cells (He and Wang 2019, Ladányi *et al*. 2022). Increased expression of HSPA1B correlates with the ability of melanoma cells to evade the immune response, leading to increased melanoma growth and poor overall survival (He and Wang 2019, Ladányi *et al*. 2022). Another gene within the HSP family, HSPB1, was selected as a top marker that instead inhibits melanoma growth. Cleaving HSPB1 into fragments has a significant effect on tumor progression, depending on the fragment (Choi *et al*. 2014). For instance, the secretion of the C-terminal HSPB1 fragment has been found to inhibit the progression of melanoma cells (Choi *et al*. 2014). CD63 also acts as a suppressor of tumor progression, as elevated expression decreases melanoma cell motility and invasiveness (Lupia *et al*. 2014).

Selected markers from the Tumor 2 cluster play key roles in the prognosis of melanoma patients. HLA-DRA, a key biomarker linked to the development and progression of skin cutaneous melanoma, ranks as the 46th most strongly associated gene with this disease (Chen *et al*. 2019, Ji *et al*. 2022). HLA-DPA1 was found to be the 80th most closely correlated gene with cutaneous melanoma (Chen *et al*. 2019). Survival analysis showed that increased expression of HLA class II genes, particularly HLA-DP and HLA-DR, in cutaneous melanoma was strongly linked to improved overall survival rates (Chen *et al*. 2019). CD74, a gene that works in conjunction with HLA-DP and HLA-DR genes, is important in the development of adaptive immune responses (Chen *et al*. 2019, Liu *et al*. 2020). It has also been associated with improved prognosis in melanoma patients (Chen *et al*. 2019, Liu *et al*. 2020). This suggests that these selected marker genes may play a critical role in enhancing the immune response against melanoma, contributing to improved patient outcomes. On the other hand, the expression of one selected marker, GADD45B, plays a critical role in the melanoma cell cycle (Chen *et al.* 2019, 2023). Down-regulation of this gene can cause accelerated transitions between melanoma cell cycle phases, accelerating tumor growth (Chen *et al*. 2019, 2023). CORTADO selected markers that implicate either positive or negative prognosis for melanoma patients, showcasing the model’s use in determining the overall survival of skin cancer patients.

Figure 6A shows a protein-protein interaction (PPI) network analysis for tumor biomarkers in basal cell carcinoma to report significant connectivity among nine key proteins (GADD45B, HSPA1B, HSPB1, CD63, CST3, CD74, HLA-DRA, HLA-DPA1, and NPC2), with a total of 16 interactions identified (*P*-value = 9.14e−07) (Ge *et al*. 2020, Szklarczyk *et al*. 2023). The PPI network was constructed using ShinyGo (Ge *et al*. 2020). The observed number of interactions far exceeded the expected random interactions (n=3), highlighting a significant enrichment of functional associations within this biomarker set. Notably, CD74 emerged as a central hub in the network, interacting with multiple proteins, including HLA-DRA and HLA-DPA1, which are key mediators of antigen presentation and immune response regulation (Crux and Elahi 2017). Other proteins, such as GADD45B and HSPB1, are known to be involved in stress response and cellular homeostasis, which are critical in tumorigenesis and cancer progression (Tamura *et al*. 2012). These findings suggest that the identified proteins are not only interrelated but may also collectively contribute to basal cell carcinoma pathophysiology, providing insights into potential therapeutic targets.

The pathways enriched in genes upregulated in tumor-marker cells from the basal cell carcinoma (BCC) cluster primarily involve antigen processing, presentation, and immune activation. These findings provide insights into the interactions between tumor cells and immune cells within the BCC microenvironment (Yost *et al*. 2019). The list of pathways enriched is in Tables 1 and 2. We sorted the pathways that met the .05 cut-off in increasing order of their *P*-adjusted values. The upregulation of pathways such as myeloid dendritic cell antigen processing and presentation, antigen processing and presentation via MHC class II, and MHC class II protein complex assembly highlights the role of antigen-presenting cells (APCs), such as dendritic cells, in processing and presenting tumor-associated antigens (Ten Broeke *et al*. 2013). These antigens are likely derived from neoantigens generated by UV-induced DNA damage, which is a hallmark of BCC (de Gruijl *et al*. 2001), suggesting that tumor cells and associated APCs actively participate in eliciting an anti-tumor immune response by engaging immune cells through MHC class II pathways.

**Table 1:** Top 10 enriched GO pathways with adjusted *P*-values for the Tumor 1 cluster.

GO term	Description	Adjusted *P*-value
GO:0001895	retina homeostasis	0.002733921
GO:0070370	cellular heat acclimation	0.018137037
GO:0070432	regulation of nucleotide-binding oligomerization domain containing 2 signaling pathway	0.018137037
GO:0046836	glycolipid transport	0.018137037
GO:0010286	heat acclimation	0.018137037
GO:0099641	anterograde axonal protein transport	0.018137037
GO:1905383	protein localization to presynapse	0.018137037
GO:0038089	positive regulation of cell migration by vascular endothelial growth factor signaling pathway	0.018137037
GO:0090063	positive regulation of microtubule nucleation	0.018137037
GO:1903265	positive regulation of tumor necrosis factor-mediated signaling pathway	0.018137037

**Table 2:** Top 10 enriched GO pathways with adjusted *P*-values for the Tumor 2 cluster.

GO term	Description	Adjusted *P*-value
GO:0016064	Immunoglobulin Mediated Immune Response	2.34e-6
GO:0002503	Peptide Antigen Assembly With MHC Class II Protein Complex	1.22e-4
GO:0002399	MHC Class II Protein Complex Assembly	1.22e-4
GO:0002381	Immunoglobulin Production Involved In Immunoglobulin-Mediated Immune Response	1.33e-4
GO:0002501	Peptide Antigen Assembly With MHC Protein Complex	1.33e-4
GO:0019886	Antigen Processing And Presentation Of Exogenous Peptide Antigen Via MHC Class II	2.19e-4
GO:0002495	Antigen Processing And Presentation Of Peptide Antigen Via MHC Class II	2.19e-4
GO:0002478	Antigen Processing And Presentation Of Exogenous Peptide Antigen	2.82e-4
GO:1903039	Positive Regulation Of Leukocyte Cell-Cell Adhesion	5.05e-4
GO:0051251	Positive Regulation Of Lymphocyte Activation	5.24e-4

We also note enrichment in pathways identified by CORTADO, namely, positive regulation of T-cell activation and positive regulation of leukocyte cell-cell adhesion. This indicates that immune cell recruitment and activation are prominent processes in the tumor microenvironment (TME) (Giraldo *et al*. 2019). This is consistent with the immune-visible nature of BCC, where immune cells infiltrate the tumor to mount a response (Zilberg *et al*. 2023). The broader pathway enrichment in positive regulation of multicellular organismal processes reflects the systemic impact of localized immune responses within the TME (Giraldo *et al*. 2019). Signals from tumor cells may propagate to influence systemic immunity or enhance local immune modulation, balancing pro-inflammatory and immune evasion dynamics.

These findings align with the known biology of BCC, a tumor type characterized by its interaction with the immune system due to chronic mutagenic exposure (de Gruijl *et al*. 2001). While immune infiltration and antigen presentation are evident, tumor cells may evade immune clearance through mechanisms such as altered antigen processing, secretion of immunosuppressive cytokines, or checkpoint pathway activation (Ten Broeke *et al*. 2013).

We performed additional case-studies showcasing CORTADO’s effectiveness on real-world datasets. In Supplementary Materials Section 2 we ran CORTADO on the Zeisel Mouse Brain dataset, and find novel biomarkers for the Pyramidal CA1 cells (Zeisel *et al*. 2015). Additionally in Supplementary Materials Section 3 we showcase CORTADO’s ability to identify markers in FCGR3A+ cells within a PBMC dataset (Hoffman 2024).

## Discussion

We introduce CORTADO, a novel method for identifying marker genes in scRNA-seq data and improving clustering accuracy through iterative refinement. Unlike traditional approaches that rely solely on statistical tests or correlation metrics, CORTADO combines differential expression analysis with cosine similarity optimization to identify robust, non-redundant, and biologically meaningful markers. Its unique strength lies in its ability to directly link marker selection to downstream clustering, exhibiting dual utility as both a marker discovery framework and a clustering enhancement tool.

Our benchmarking experiments highlight several key findings. First, CORTADO consistently outperformed existing methods in selecting genes with the highest differential expression while also ranking second-best for minimizing redundancy (in terms of cosine similarity), reflecting its ability to balance expression specificity with marker diversity. Unlike methods that excel at only one metric, CORTADO demonstrated robust and stable performance across both, making it a reliable approach for diverse datasets. Second, by incorporating CORTADO-selected markers into an iterative refinement framework, we showed that clustering accuracy substantially improved across multiple datasets, with results surpassing both COSG and standard Leiden clustering based on highly variable genes. This underscores the value of marker optimization not just for interpretation, but also as a practical driver of cell-type separability.

We further validated CORTADO across a variety of biological contexts. In the Zeisel mouse brain dataset, CORTADO identified neuron-specific markers that were overlooked by other methods yet had functional relevance. In the DLPFC spatial transcriptomics dataset, CORTADO-selected markers exhibited stronger spatial localization than those from COSG or Wilcoxon tests, directly linking marker identity to tissue structure. In the PBMC3K dataset, CORTADO successfully retrieved canonical immune cell markers with clinical importance, and in the basal cell carcinoma dataset, it identified tumor-associated markers enriched in immune pathways, validated through pathway enrichment and PPI analysis, demonstrated in the Supplementary Materials. Together, these results demonstrate that CORTADO not only produces markers with computational robustness but also uncovers biologically and clinically relevant insights.

The average runtime of CORTADO is around 4.5 s for a dataset with around 10 clusters; the runtime decreases as the number of clusters is decreased. In the Supplementary Materials Fig. S8 (in Supplementary Materials Section 4), we perform a sensitivity analysis on the Zeisel Mouse Brain dataset by varying hyperparameters of CORTADO and comparing runtime (Zeisel *et al*. 2015). The results of this figure demonstrate that changing the parameters of CORTADO does not affect the runtime drastically. Further optimizations to the CORTADO method are possible, such as parallel processing of each cluster or optimizing the number of neighbors calculated within the hill-climbing algorithm. We plan to investigate ways to improve the CORTADO runtime in future work.

We are considering several future extensions. One direction involves incorporating multi-omics data, such as DNA methylation or chromatin accessibility, to capture additional regulatory dimensions of marker genes. Another is refining the initial filtering step: while CORTADO currently emphasizes highly differentially expressed genes, integrating dimensionality reduction techniques could allow the discovery of subtle but biologically meaningful markers that may not pass conventional thresholds. Finally, expanding the iterative refinement strategy to integrate with advanced clustering frameworks could further improve the resolution of rare or transitional cell states.

## Conclusion

CORTADO advances marker gene discovery and clustering analysis in single-cell transcriptomics through a unified optimization framework. By maximizing differential expression, minimizing redundancy via cosine similarity, and enforcing marker set sparsity, CORTADO reliably identifies robust and non-redundant markers across diverse datasets. Beyond marker discovery, CORTADO’s iterative refinement procedure demonstrates that these markers directly improve clustering accuracy, improving Scanpy clustering by a margin larger than COSG. This dual contribution establishes CORTADO as a tool that not only identifies meaningful molecular signatures but also enhances the fundamental process of cell-type resolution.

With its strong performance, adaptability, and demonstrated utility in both marker selection and clustering improvement, CORTADO represents a significant advancement in single-cell transcriptomics. Future extensions toward multi-omics integration and advanced clustering strategies promise to expand its scope further, positioning CORTADO as a versatile framework for uncovering cellular heterogeneity and guiding biological discovery.

## Methods

### Preprocessing

First, a single-cell RNA sequencing (scRNA-seq) dataset was loaded into Python using the scanpy package and stored as an AnnData object. Low-quality cells were filtered based on gene count thresholds, and cells with high mitochondrial gene expression were removed to exclude dying cells. Genes expressed in fewer than 5 cells are discarded. Library size normalization was performed using sc.pp.normalize_total() (defaulting to 10,000 counts per cell), followed by log transformation with sc.pp.log1p() to stabilize the variance. For all methods, we only considered the top 2000 highly variable genes using the sc.pp.highly variable genes() function in Scanpy (Wolf *et al*. 2018). These preprocessing steps were performed on all datasets used in this paper for each method, except for methods that had their own preprocessing methods, such as scMAGS (Baran and Doğan 2023). In those instances, we followed the author’s recommended guidelines.

### CORTADO based iterative clustering refinement algorithm

Algorithm 1 Iterative Marker-Clustering Refinement

**Require:** Single-cell data matrix X, convergence threshold τ, maximum iterations *M*

**Ensure:** Final cluster labels, marker genes, refined gene pool

1: **Initialize:** 

2: G←highly_variable_genes(X,n_top)

3: $l_{\mathrm{prev}}\leftarrow \text{NULL}$

4: k←1

5: **repeat** 

6:  **//STEP 1: CLUSTERING** 

7:  $l_{\mathrm{current}}\leftarrow \text{cluster}\_\text{cells}(X,G)$:

8:   ▹ Subset data to gene pool *G*

9:   ▹ Run pipeline: normalize → log → scale → PCA → neighbors → Leiden

10:  **//CHECK CONVERGENCE** 

11:  **if**  $l_{\mathrm{prev}}\neq \text{NULL}$  **then** 

12:   $\text{ARI}\leftarrow \text{adjusted}\_\text{rand}\_\text{score}(l_{\text{prev}},l_{\text{current}})$

13:   **if**  ARI≥τ  **then** 

14:    **break** 

15:   **end if** 

16:  **end if** 

17:  **//STEP 2: MARKER SELECTION** 

18:  $M_{\text{clusters}}\leftarrow \text{find}\_\text{markers}\_\text{per}\_\text{cluster}(X,l_{\text{current}},\text{method})$:

19:   ▹ For each cluster: find top markers using CORTADO or COSG

20:   ▹ Skip clusters with too few cells

21:  **//UPDATE GENE POOL** 

22:  $G\leftarrow \underset{\text{all}\;\text{clusters}}{\cup }M_{\text{clusters}}$

23:  $l_{\mathrm{prev}}\leftarrow l_{\mathrm{current}}$

24:  k←k+1

25: **until** convergence OR k>M

26: **return**  $l_{\mathrm{current}}$, $M_{\text{clusters}}$, *G*

Algorithm 2 Stochastic Hill Climbing (CORTADO)

**Require:** Marker scores, similarity matrix, parameters γ, *T*, *L*, *K*, *k*

**Ensure:** Optimal gene subset $X^{*}$

1: Initialize binary solution $X_{0}$ with *k* selected genes

2: $X^{*}\leftarrow X_{0}$,  t←0,  idle←0

3: **while**  t<T  **and**  idle<L  **do** 

4:  Compute exploration rate $\epsilon_{t}\leftarrow \gamma^{t}$

5:  Generate *K* neighboring solutions via random bit-flip

6:  **if**  $\text{Uniform}(0,1)<\epsilon_{t}$  **then**    ▹ Explore

7:   Move to a randomly selected neighbor

8:  **else**        ▹ Exploit

9:   **if** any neighbor improves objective **then** 

10:    Move to best improving neighbor; update $X^{*}$; reset idle

11:   **else** 

12:    Stay; increment idle counter

13:   **end if** 

14:  **end if** 

15:  t←t+1

16: **end while** 

17: **return**  $X^{*}$

### Stochastic-Hill optimization

#### Constrained and unconstrained hill-climbing optimization

The hill-climbing optimization employed by CORTADO applies a stochastic approach tailored for selecting a near-optimal subset of genes from a given set. Each element in the binary vector *X* represents a gene, where a value of $X_{g}=1$ indicates that the gene *g* is selected, and 0 means it is not. CORTADO begins with an initial solution and iteratively explores the neighborhood of the current solution to identify better configurations (as per the optimization goals defined next). At each step, new candidate solutions (or “neighbors”) are generated by flipping one or more bits in the binary vector, and their fitness is evaluated based on a predefined objective function. An adaptive exploration mechanism, where the exploration rate decreases exponentially over iterations (denoted by a time variable *t*) by a factor of γ∈[0,1], balances the need to escape local optima with the focus on refining the solution. The optimization terminates if no improvement is observed for a defined number of steps *L*.

Notably, CORTADO handles both *constrained* and *unconstrained* scenarios for gene selection. In the unconstrained case, *X* is initialized randomly, allowing any combination of genes to be selected without restrictions. In contrast, the constrained case ensures that *X* always contains a fixed number of genes selected (i.e. an exact number of 1 s). This constraint is maintained during the generation of neighboring solutions, where any bit-flipping operation must preserve the total count of selected genes. The constrained approach adds complexity but ensures that the optimization process aligns with user-defined scenarios requiring the selection of a predefined number of genes.

#### Optimization goals

The optimization process for marker gene selection is guided by an objective function with three components, each targeting a specific goal: maximizing the differential marker score within the cluster of interest versus other clusters, minimizing overlap in the expression profiles of selected marker genes, and minimizing the number of selected genes (i.e. sparseness of the solution). The overall objective function is expressed as:


$$\text{Objective:}\lambda_{1}c_{1}-\lambda_{2}c_{2}-\lambda_{3}c_{3}$$

Here, the components $c_{1}$, $c_{2}$, and $c_{3}$ are defined as follows:


$$c_{1}=\sum_{g=1}^{n_{\text{Genes}}}X_{g}\cdot \text{marker}\_{\text{score}}_{g}$$

$$c_{2}=-2\frac{\sum_{g_{1}=1}^{n_{\text{Genes}}-1}\sum_{g_{2}=g_{1}+1}^{n_{\text{Genes}}}X_{g_{1}}\cdot X_{g_{2}}\cdot \text{sim}\_{\text{score}}_{g_{1},g_{2}}}{\left(\sum_{g=1}^{n_{\text{Genes}}}X_{g}\right)\left(\left(\sum_{g=1}^{n_{\text{Genes}}}X_{g}\right)-1\right)+1}$$

$$c_{3}=\sum_{g=1}^{n_{\text{Genes}}}X_{g}$$

#### Maximizing differential marker scores ($c_{1}$)

The term $c_{1}$ prioritizes the selection of genes with a strong differential expression between the cluster of interest and other clusters. For a gene *g*, the marker score is:


$$\text{marker}\_{\text{score}}_{g}=\frac{1}{G}\sum_{g=1}^{G}{\;\text{log}\;}_{2}\left(\frac{{\text{DE}\;}_{\text{target}}(g)}{\text{mean}\left[{\text{DE}}_{\text{other}}(g)\right]}\right)$$

In this equation:

- *G* is the total number of genes in the dataset (while *n* is a model input representing the number of genes to be selected).
- DE factor in the cluster of interest refers to the absolute value of the log${\;}_{2}$ fold-change in expression of gene *g* between the cluster of interest and a baseline.
- Mean DE factor in all other clusters is the average absolute log${\;}_{2}$ fold-change of gene *g* across all clusters except the one of interest.
- The ${\;\text{log}\;}_{2}$ transformation emphasizes relative changes and ensures fold-changes are measured on a symmetric scale around zero. We use Min Max scaling to ensure that all marker score values are scaled between 0 and 1.
- The entire formula computes the average log${\;}_{2}$ fold-change of selected genes, capturing how strongly each selected gene separates the target cluster from others.

By summing these marker scores weighted by the binary variable $X_{g}$ (where $X_{g}=1$ if the gene is selected, 0 otherwise), this component of the objective function ensures that selected genes provide maximum discrimination for the cluster of interest.

#### Minimizing overlap in expression profiles ($c_{2}$)

The cosine similarity term $c_{2}$ penalizes the selection of gene pairs with high similarity in their expression profiles, promoting diversity among the selected marker genes. It is defined as:


$$c_{2}=-2\frac{\sum_{g_{1}=1}^{n_{\text{Genes}}-1}\sum_{g_{2}=g_{1}+1}^{n_{\text{Genes}}}X_{g_{1}}\cdot X_{g_{2}}\cdot \text{sim}\_{\text{score}}_{g_{1},g_{2}}}{\left(\sum_{g=1}^{n_{\text{Genes}}}X_{g}\right)\left(\left(\sum_{g=1}^{n_{\text{Genes}}}X_{g}\right)-1\right)+1}$$

The $\text{sim}\_{\text{score}}_{g_{1},g_{2}}$ represents the cosine similarity between the expression profiles of genes $g_{1}$ and $g_{2}$. The term is scaled by $\lambda_{1}$, which determines the relative weight of diversity. By dividing by the number of selected gene pairs, the penalty is normalized, ensuring fair contribution regardless of the total number of selected genes.

#### Encouraging sparseness in gene selection ($c_{3}$)

The selection penalty term $c_{3}$ discourages the inclusion of too many genes, promoting sparseness in the final selection. It is defined as:


$$c_{3}=\sum_{g=1}^{n_{\text{Genes}}}X_{g}$$

Here, $\lambda_{2}$ controls the penalty strength, with higher values favoring smaller sets of marker genes. This term ensures that the optimization process prioritizes compact and efficient gene sets, which are easier to interpret and validate biologically.

#### Optimization strategy

The optimization is performed using an iterative procedure that balances these three components. The weights $\lambda_{1}$, $\lambda_{2}$, and $\lambda_{3}$ are chosen based on cross-validation, ensuring the resulting gene sets meet the goals of *specificity*, *diversity*, and *sparseness*. By scaling the marker score between 0 and 1, all individual components are weighted similarly, and one of them cannot dominate the other aspects of the objective function. This approach is particularly effective in single-cell studies, where a small set of discriminative marker genes helps downstream analysis and experimental validation. In Table 3 we display all other hyperparamters of CORTADO that are customizable by the user, including the default parameters that we recommend.

**Table 3:** CORTADO hyperparameters and their default values.

Name	Type	Default value	Description
$\lambda_{1}$	float	0.9	Weight on differential expression reward term
$\lambda_{2}$	float	0.1	Weight on redundancy (cosine similarity) penalty
$\lambda_{3}$	float	0.0	Weight on sparsity/marker count penalty
exploration${\;}_{\text{rate}}$	float	0.95	Probability of accepting exploratory (non-greedy) moves
idle${\;}_{\text{rate}}$	int	10	Maximum iterations allowed without improvement before stopping
max${\;}_{\text{steps}}$	int	100	Maximum number of hill-climbing iterations
constrained	bool	False	Whether to enforce a fixed number of markers
n${\;}_{\text{markers}}$	int	5	Exact number of markers (used if constrained = True)
neighbor${\;}_{\text{size}}$	int	1	Number of genes flipped per optimization step

### Benchmarking methods

#### Scanpy DE methods: Wilcoxon rank sum and *t*-test

The Scanpy package facilitates differential expression analysis in single-cell datasets, offering versatile methods to identify marker genes across clusters. We implemented differential expression using the rank_gene_groups function in Scanpy, modifying the method parameter to test with both the Wilcoxon Rank Sum test and the t-test. This flexibility allows for a comparative evaluation of these statistical approaches for detecting differentially expressed genes in single-cell data (Wolf *et al*. 2018). The analysis involves calculating the log2foldchange between the cluster of interest and other clusters. Subsequently, the chosen statistical test is applied to assign a confidence score to each gene, thereby identifying significant markers.

#### Cosg

COSG is a standard tool designed for identifying and ranking marker genes, facilitating cell-type classification across various single-cell data modalities. Supporting single-cell RNA sequencing (scRNA-seq), single-cell ATAC sequencing (scATAC-seq), and spatial transcriptomic data, COSG leverages cosine similarity to evaluate gene expression patterns (Dai *et al.*  2022). The method begins by generating an artificial gene that uniquely represents a specific cluster, ensuring it is not expressed in other cell groups. COSG then computes the cosine similarity between the representative vector of this artificial gene and the representative vector of each expressed gene in the dataset. The genes are then ranked based on their COSG scores, allowing researchers to systematically identify high-confidence markers critical for cell-type classification and downstream analyses.

#### scMAGS

scMAGS is a Python-based tool designed for identifying marker genes that define specific cell clusters within single-cell RNA sequencing (scRNA-seq) data. It focuses on selecting genes that best characterize the distinctiveness of cell clusters. The method begins with a cluster-specific gene filtering step, narrowing the pool of potential marker genes by evaluating their relevance to individual clusters. To ensure robustness, scMAGS utilizes quantitative metrics tailored to the dataset’s size and structure. For smaller datasets, the Silhouette index is employed to measure how well a sample aligns with its cluster relative to others. For larger datasets, the Calinski-Harabasz index is used, leveraging its ability to assess the ratio of inter-cluster to intra-cluster similarity. This dual approach is intended to allow scMAGS to adapt effectively to varying dataset characteristics (Baran and Doğan 2023).

#### RankCorr

RankCorr is a Python-based tool designed for multi-class marker selection, specifically for single-cell RNA sequencing (scRNA-seq) data. It finds marker genes that distinguish between different cell types or classes (Vargo and Gilbert 2020). It ranks the input mRNA count data, converting it into a ranked representation that highlights relative expression levels across genes. Linear separation techniques are then applied to identify distinct patterns within the ranked data, enabling the differentiation of cell types. To ensure adaptability, RankCorr determines the optimal number of markers based on the dataset’s characteristics.

## Supplementary Material

vbag106_Supplementary_Data

## Data Availability

All datasets used for benchmarking are publicly available. Table 4 below lists the names of each dataset and where they can be accessed. Datasets and their corresponding databases with accession numbers or links.
