# Supplementary material for: CORTADO: hill climbing optimization for cell-type specific marker gene discovery and clustering accuracy improvement
Source: Bioinform Adv. 2026 Apr 13;6(1):vbag106. doi: 10.1093/bioadv/vbag106 (PMC13273416; doi:10.1093/bioadv/vbag106)
Supplement: vbag106_Supplementary_Data [file vbag106_supplementary_data.pdf]

CORTADO: Hill Climbing Optimization for Cell-Type Specific Marker Gene Discovery and Clustering Accuracy Improvement: Supplementary Materials

Musaddiq K. Lodi[1], Leiliani Clark[2], Satyaki Roy[3], and Preetam Ghosh[4,*]

[1]Integrative Life Sciences, Virginia Commonwealth University, Richmond, VA, USA

[2]Center for Biological Data Science, Virginia Commonwealth University, Richmond, VA, USA

[3]Department of Mathematical Sciences, University of Alabama in Huntsville, Huntsville, AL, USA

[4]Department of Computer Science, Virginia Commonwealth University, Richmond, VA, USA

Supplementary Materials

1 Sensitivity Analysis

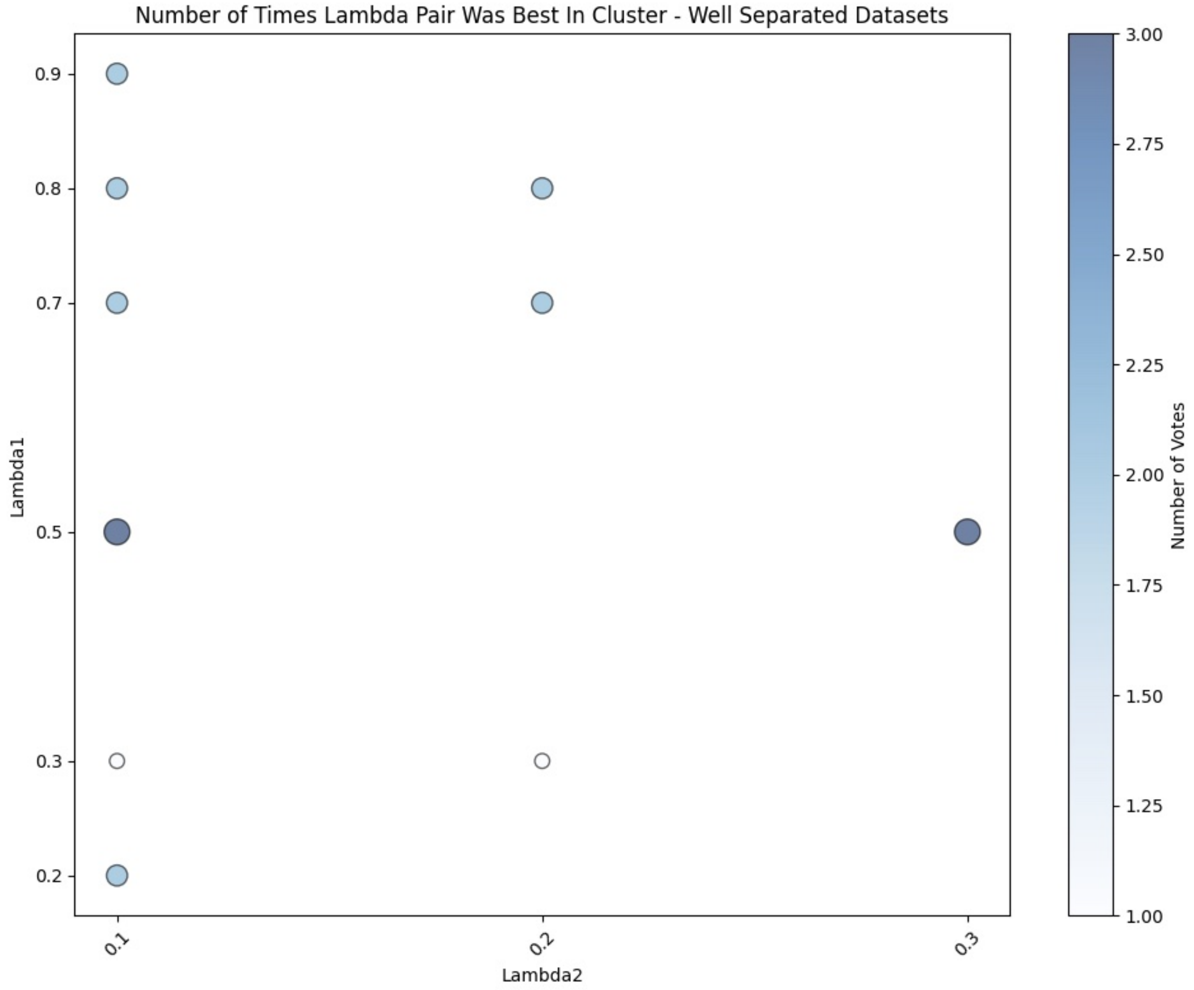

Figure 1: Sensitivity analysis for well-separated clusters.

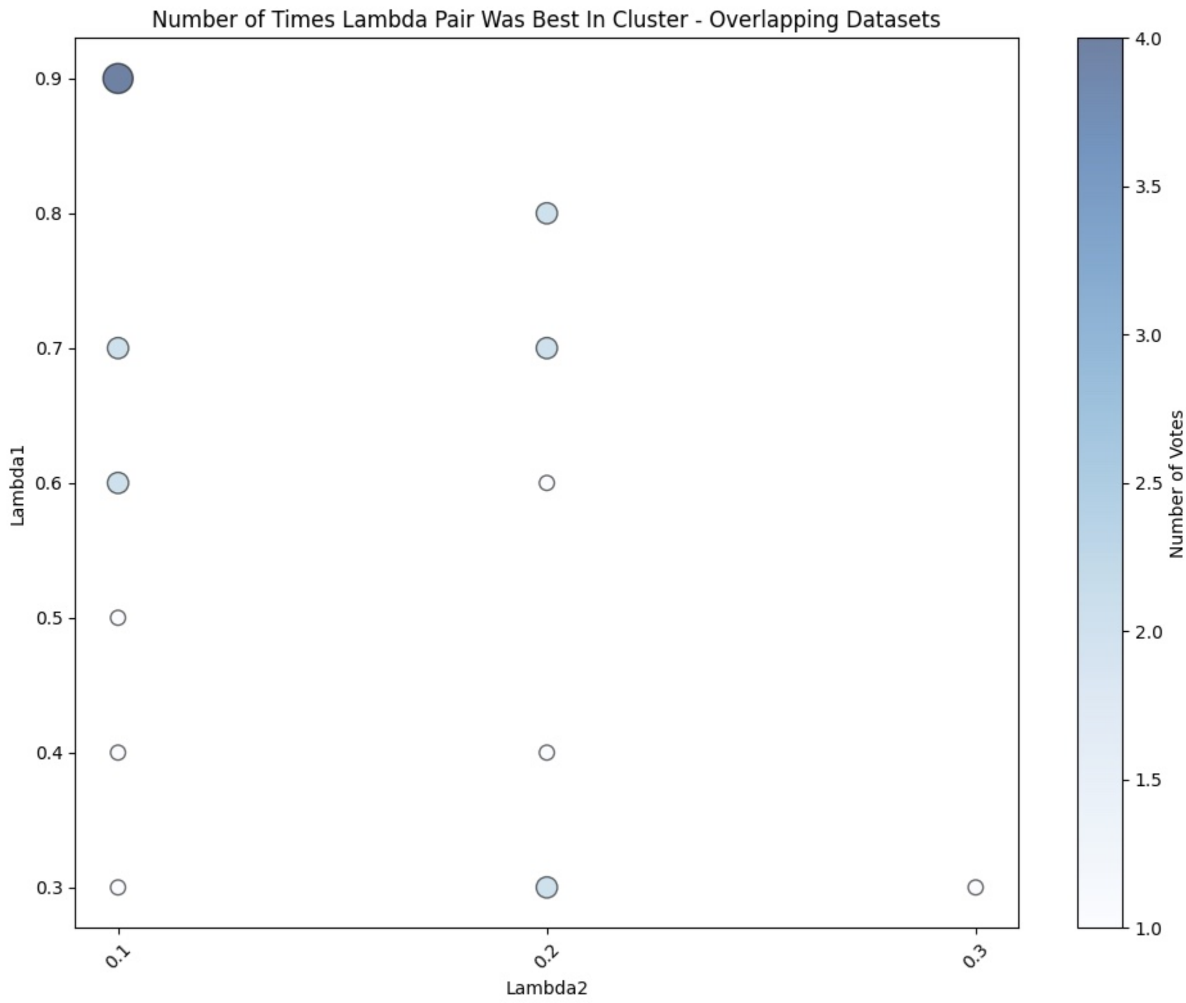

Figure 2: Sensitivity analysis for overlapping clusters

The sensitivity analysis was performed using simulated data from the Splatter package. We set up two experiments, one to determine the best λ_1 and λ_2 combination for overlapping clusters, and another to determine the best λ_1 and λ_2 combination for well-separated clusters. The well-separated and overlapping clusters were generated by adjusting Splatter parameters, specifically by increasing the `de.prob`, `de.FacLoc`, and `de.facScale` parameters in the overlapping cluster dataset, using the function `setParams` [1].

We evaluated by calculating the difference in log2foldchange in markers in the cluster of interest versus all other clusters, for every cluster across simulated datasets. Figure 1 demonstrates that the best λ_1 and λ_2 combination for well-separated clusters is 0.5 and 0.3 respectively; Figure 2 shows that the best λ_1 and λ_2 combination for overlapping clusters is 0.9 and 0.1 respectively. For the downstream experiments and benchmarking

evaluations, we used the overlapping parameters of 0.9 and 0.1 as this would more closely resemble real scRNAseq data.

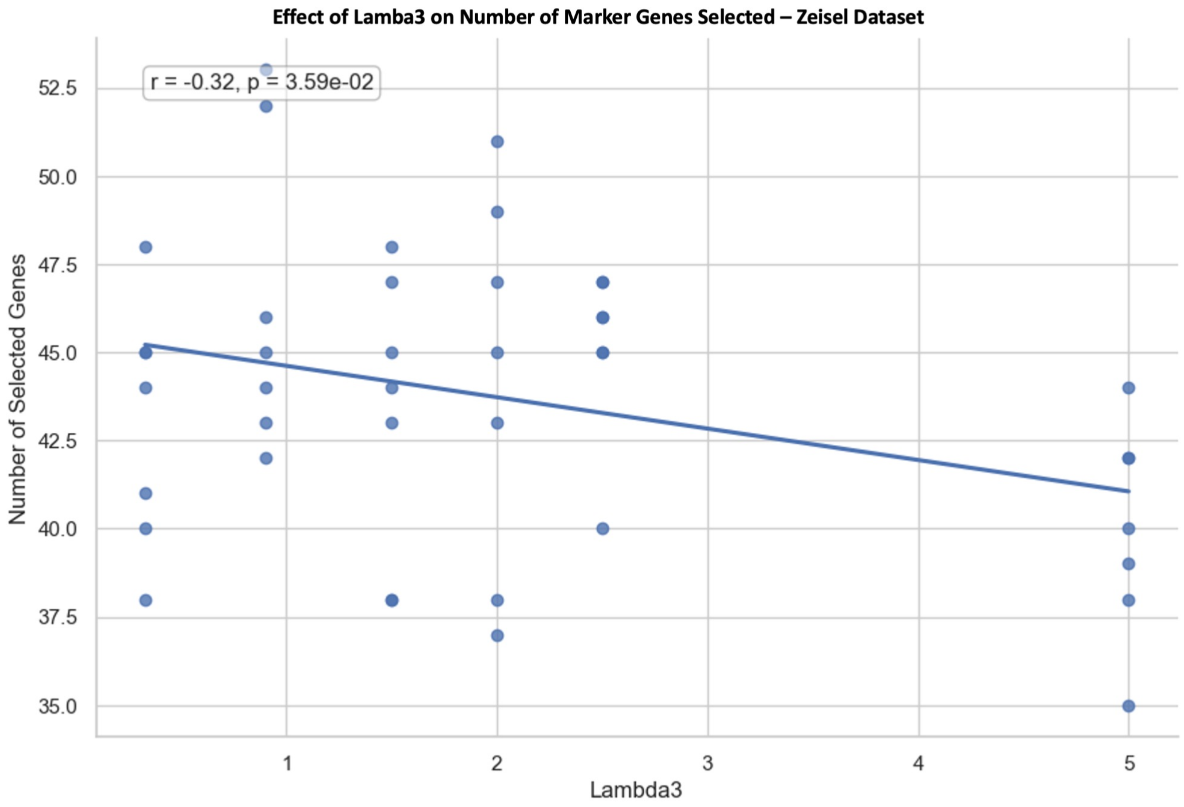

Figure 3: Effect of increasing λ_3 on the number of selected genes in the Zeisel Mouse Brain dataset.

For the unconstrained version of CORTADO, we evaluated the effect of increasing λ_3 on the number of marker genes selected. We chose to observe this effect on the Zeisel Mouse Brain dataset, as it has very well-separated clusters [2]. We see in Figure 3 that

increasing the λ_3 value decreases the number of genes selected by CORTADO. We fixed the λ_1 and λ_2 values at 0.33 increased$\lambda 3$, running several of these combinations across all clusters in the dataset. This experiment verifies the sensitivity of the model to λ_3, and allows users to specify the selectivity of the model while not fixing the number of genes chosen.

2 CORTADO can distinguish high fidelity marker genes in the Zeisel Mouse Brain Dataset compared to baseline methods

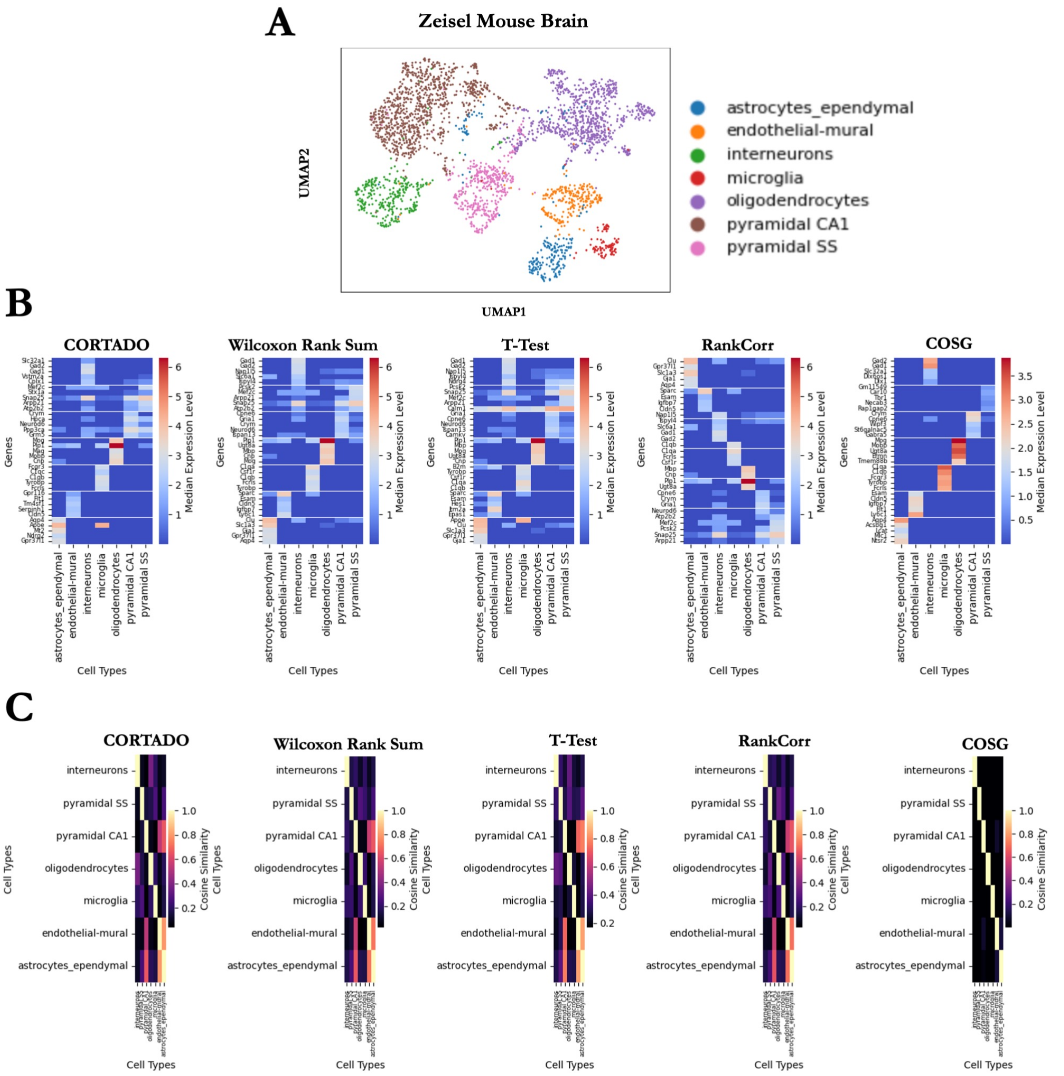

Figure 4: CORTADO analysis on the Zeisel Mouse Brain Dataset. A) Median expression level for top 5 selected markers per method across cell types. B) Cosine similarities between cell-type median gene expression for the top 5 selected markers per method. C) UMAP Visualization for cell types in the Zeisel Mouse Brain dataset.

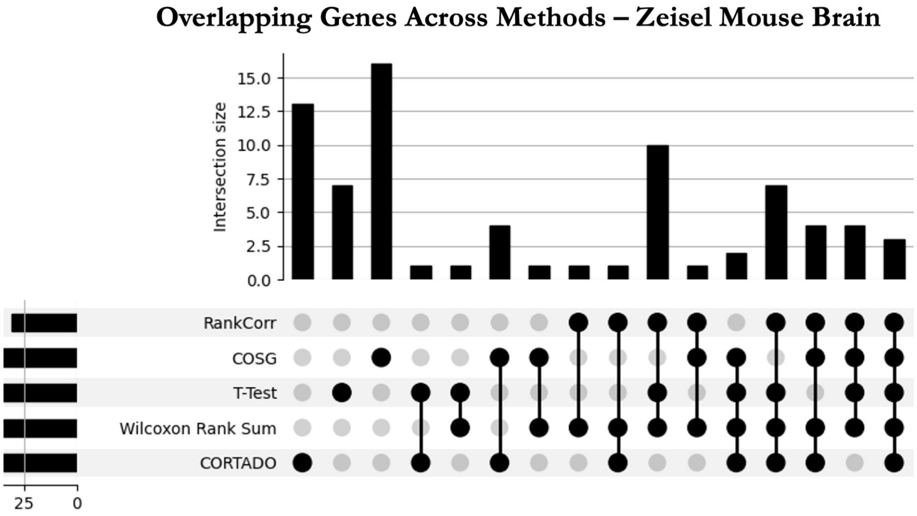

Figure 5: Upset plot depicting overlapping genes selected for each method on Zeisel Mouse Brain Dataset. The horizontal bars on the left represent the total number of genes identified by each method individually, while the vertical bars above indicate the size of the intersections, showing the number of overlapping genes shared among specific combinations of methods. The dots represent the methods included in each intersection, with connected lines linking methods that share genes. The number of overlapping genes for each combination is denoted in the bargraph at the top of the figure.

GO Term	Description
GO:0019722	Calcium-Mediated Signaling
GO:0050804	Modulation of Chemical Synaptic Transmission
GO:0010359	Regulation of Anion Channel Activity
GO:0060993	Kidney Morphogenesis
GO:0043403	Skeletal Muscle Tissue Regeneration
GO:0046877	Regulation of Saliva Secretion
GO:0090184	Positive Regulation of Kidney Development
GO:1905665	Positive Regulation of Calcium Ion Import Across Plasma Membrane
GO:0048741	Skeletal Muscle Fiber Development
GO:0070262	Peptidyl-Serine Dephosphorylation
GO:0033173	Calcineurin-NFAT Signaling Cascade
GO:0097205	Renal Filtration
GO:1905664	Regulation of Calcium Ion Import Across Plasma Membrane
GO:0051047	Positive Regulation of Secretion
GO:0097720	Calcineurin-Mediated Signaling
GO:0014904	Myotube Cell Development

Table 1: Enrichment Analysis Results with GO Terms and Descriptions for Selected Markers Zeisel Mouse Brain Dataset: Pyramidal CA1 Cells

The Zeisel mouse brain dataset contains 3005 cells from mouse hippocampus tissue, with 7 distinct clusters [2]. This dataset is commonly used for scRNAseq benchmarking studies, as the clusters are biologically relevant and well separated [3], making it relevant

for evaluating marker selection methods. The heatmap in Figure 4B depicts the expression of the top 5 selected marker genes for each cluster across all methods. This result is a very clear indicator of genes that may have high expression in more than one cell type. While it is improbable to expect that selected markers will only have expression in the cluster of interest, strong markers should generally have high expression in its intended cluster, and low expression in all other clusters. While COSG and RankCorr select the most distinct genes based on the marker gene score, CORTADO follows a similar pattern by selecting well distinguished genes, particularly in the astrocyte-ependymal, endothelial-mural, and microglia clusters. From this analysis, we see in 4A that the markers selected by CORTADO, RankCorr, and COSG follow a pattern where the markers selected have high expression in one cluster, and negligible expression in other clusters. The Scanpy implementations of the Wilcoxon Rank Sum test and t-test appear to have selected markers that have consistent expression in multiple clusters [4]. COSG in particular selected genes that are distinguished in their cluster by expression value.

The heatmap in Figure 4C shows the cosine similarity between markers selected in each method across clusters. The cosine similarity for the heatmap was calculated by averaging the expression across markers for each cluster and then computing the cosine similarity of the average expression vector. Strong markers selected by cosine similarity separation have low cosine similarity across clusters. CORTADO and COSG select markers with low cosine similarity across clusters, while other methods choose markers with high cosine similarity. The results in Figure 4B and Figure 4C confirm these findings from the quantitative benchmarking in a practical use case.

In the following experiment, we elucidate CORTADO's capability to identify unique and biologically relevant genes that are often missed by other approaches. A gene is deemed *unique* if it is selected by only a single approach for a given cluster. Figure 5 shows that CORTADO and COSG select the highest number of unique genes aggregated across clusters. To verify their biological relevance, we used the Pyramidal CA1 cell cluster. Pyramidal CA1 cells are a type of neuron that processes sensory and motor cues [5]. Pyramidal CA1 neurons are also the most abundant neurons in the brain [6]. The

unique genes selected by CORTADO to be markers for the Pyramidal CA1 cluster were Grm5, Hpca, and Ppp3ca. Furthermore, we ran a GO Biological Process (BP) enrichment using the EnrichR online tool [7] [8]. We down-selected significant pathways based on FDR Corrected p-value satisfying a 0.05 cutoff threshold, and sorted by p-value after applying the cutoff. The results of this analysis are depicted in the bar plot in Table 1. We see from this figure that the pathways being selected are related to signaling overall. Of particular interest is the Modulation of the Chemical Synaptic Transmission pathway; chemical synaptic transmission is imperative to neuronal communication, as this pathway facilitates the release of neurotransmitters in the synapse [9].

3 Markers uniquely selected by CORTADO have functional pertinence to immune system cells in peripheral blood mononuclear cell

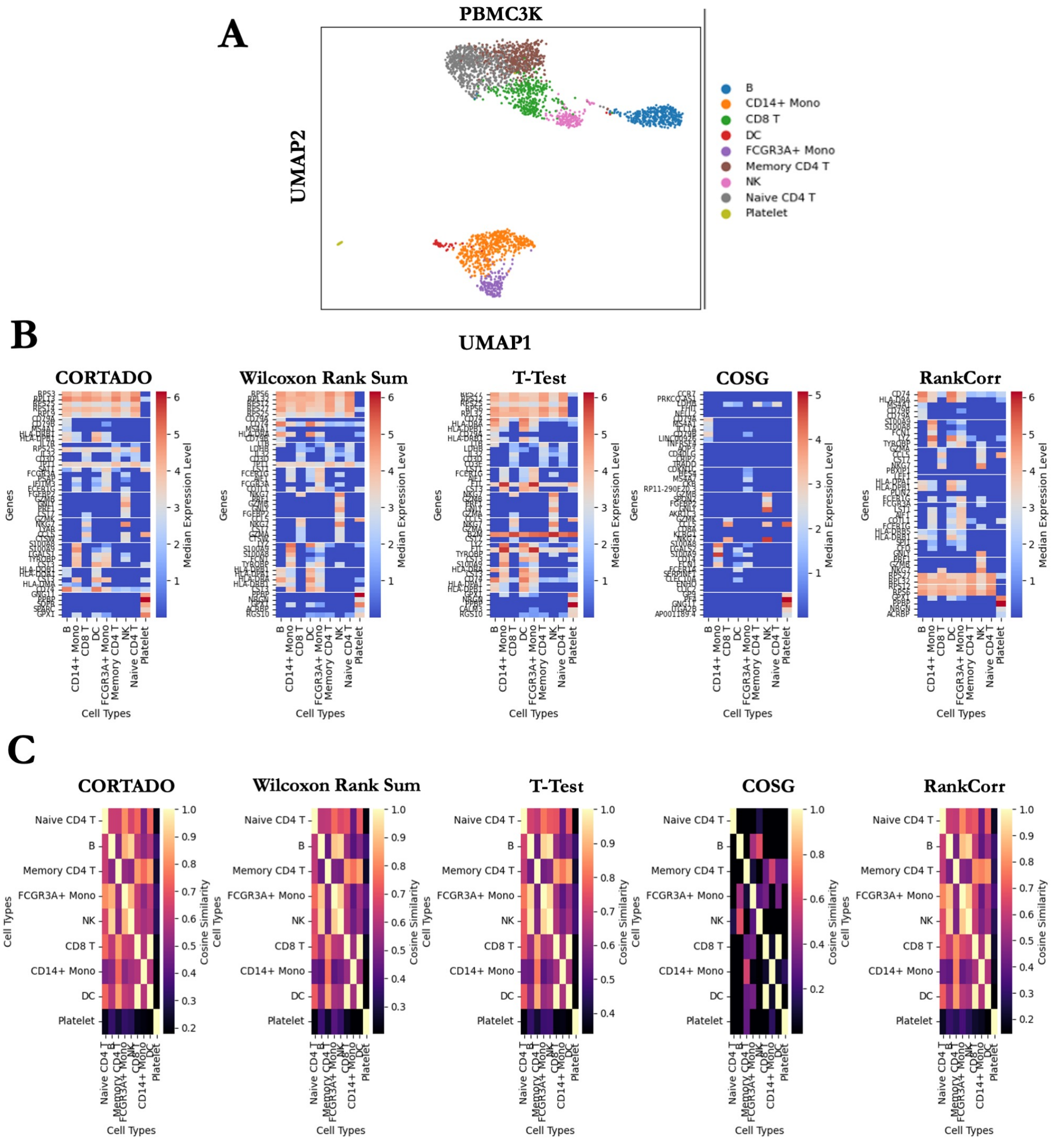

Figure 6: CORTADO analysis on the PBMC3K Dataset. A) UMAP Visualization for PBMC3K Dataset. B) Median expression level for top 5 selected markers per method across cell types. C) Cosine similarities between cell-type median gene expression for the top 5 selected markers per method.

GO Term	Description
GO:0046836	Glycolipid Transport
GO:0006596	Polyamine Biosynthetic Process
GO:0008216	Spermidine Metabolic Process
GO:0042401	Biogenic Amine Biosynthetic Process
GO:0035627	Ceramide Transport
GO:0006595	Polyamine Metabolic Process
GO:0032897	Negative Regulation of Viral Transcription
GO:0035455	Response to Interferon-Alpha
GO:0046782	Regulation of Viral Transcription
GO:0046597	Negative Regulation of Viral Entry into Host Cell
GO:1903901	Negative Regulation of Viral Life Cycle
GO:0035456	Response to Interferon-Beta
GO:0080090	Regulation of Primary Metabolic Process
GO:0060337	Type I Interferon-Mediated Signaling Pathway
GO:0071357	Cellular Response to Type I Interferon

Table 2: Enrichment Analysis Results with GO Terms and Pathway Descriptions on CORTADO Markers Selected for FCGR3A+ Mono Cells

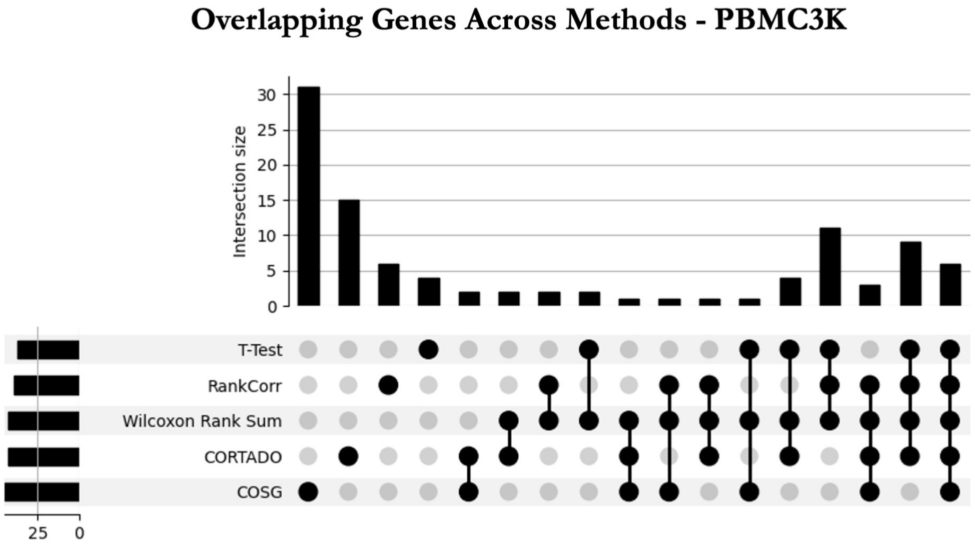

Figure 7: Upset plot of unique genes selected for PBMC3K dataset across methods. The horizontal bars on the left represent the total number of genes identified by each method individually, while the vertical bars above indicate the size of the intersections, showing the number of overlapping genes shared among specific combinations of methods. The dots represent the methods included in each intersection, with connected lines linking methods that share genes. The number of overlapping genes for each combination is denoted in the bar graph at the top of the figure.

The PBMC3K dataset is a peripheral blood mononuclear cell dataset containing 2700 cells with 9 clusters [10]. This dataset is commonly used for single-cell benchmarking

studies as PBMC cells are frequently used for scRNAseq experiments, and the clusters in this dataset are very well separated [11].

The expression of selected markers for each method across all clusters is depicted in Figure 6B. We see that most methods selected markers that are highly expressed in multiple clusters, and not just the cluster of interest. COSG in particular selected markers with seemingly low expression across clusters, even in the cluster of interest [12]. Based on these results in terms of expression, there was no clear winner for markers being selected based on expression, but CORTADO did extremely well in choosing markers for the NK and Platelet clusters. In terms of cosine similarity, we see that all methods selected genes that had similar cosine similarity profiles to other clusters. As anticipated, COSG selected genes that had the lowest cosine similarity to other clusters compared to the other methods [12]. This result is shown in Figure 6C.

To contextualize unique markers selected by CORTADO, we chose the FCGR3A+ Mono cluster as an example and contexualized our findings. The unique genes selected by each method across clusters are depicted in Figure 7. Three of the five genes selected by CORTADO for the FCGR3A+ Mono cluster were unique compared to other methods. To study if these genes were still functionally relevant, we examined them in detail and performed a GO BP pathway enrichment using the EnrichR tool [7]. We first used a cutoff threshold of FDR adjusted p-value being less than 0.05 to select significant pathways, and then ranked these pathways by p-value.

FCGR3A+ Mono cells are a kind of monocyte cell with the high expression of the FCGR3A gene. Monocytes are white blood cells that fight against microbacterial pathogens and have a role in fighting tumor metastasis [13]. The FCGRA3 gene encodes for a receptor important for natural killer (NK) cells, which assist immune cells in fighting off foreign invaders such as viral infections and tumor populations [14]. Based on this context, it is clear from the GO BP enrichment in Table 2 that the markers selected only by CORTADO have a strong correlation to immune pathways and viral response pathways. Pathways such as Regulation of Viral Transcription, Negative Regulation of Viral Entry into Host Cell, and Negative Regulation of Viral Life Cycle reinforce FCGR3A+ Mono

cells' role in fighting infection [14]. Additionally, a recent study validated the role of FCGRA3+ Monocytes in Type 1 Interferon pathways, which were also overrepresented in the GO BP enrichment [15]. This analysis demonstrates CORTADO's ability to choose relevant biomarkers for cell populations.

4 Runtime Sensitivity Analysis

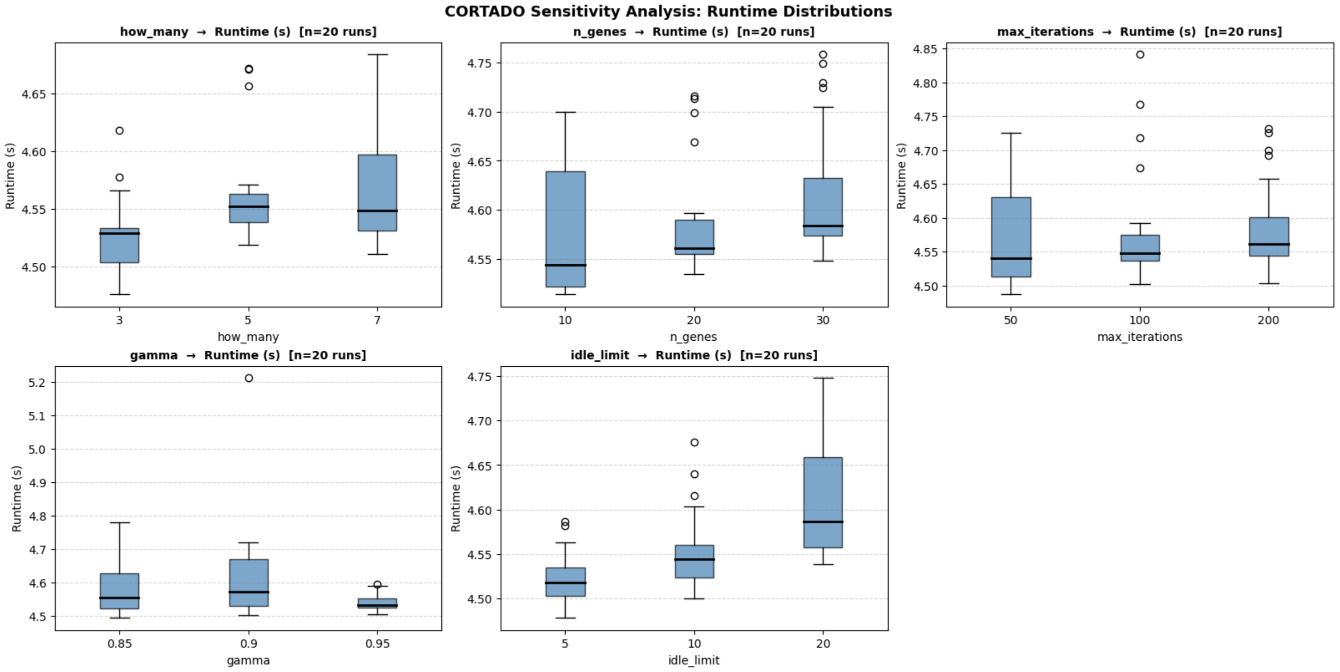

Figure 8: Sensitivity of CORTADO runtime to key hyperparameters. Boxplots show the distribution of runtime (seconds) across 20 independent runs for each tested value of six CORTADO parameters (how-many, n-genes, max-iterations, gamma, idle-limit, p-val-threshold), with all other parameters held at their defaults. The median is indicated by the black horizontal line. Wider distributions reflect greater stochasticity in the hill-climbing procedure for that configuration.

References

[1] Luke Zappia, Belinda Phipson, and Alicia Oshlack. Splatter: simulation of single-cell rna sequencing data. *Genome Biology*, 18(1):174, 2017.

[2] Amit Zeisel, Ana B. Muñoz-Manchado, Simone Codeluppi, Peter Lönnerberg, Gioele La Manno, Anna Jureus, S. Marques, H. Munguba, L. He, C. Betsholtz, K. Rolny, G. Castelo-Branco, J. Hjerling-Leffler, and Sten Linnarsson. Cell types in the mouse cortex and hippocampus revealed by single-cell rna-seq. *Science*, 347(6226):1138–1142, 2015.

[3] Alexander H. Vargo and Anna C. Gilbert. A rank-based marker selection method for high throughput scrna-seq data. *BMC Bioinformatics*, 21(1), Oct 2020.

[4] F. Alexander Wolf, Philipp Angerer, and Fabian J. Theis. Scanpy: Large-scale single-cell gene expression data analysis. *Genome Biology*, 19(1), Feb 2018.

[5] Alex R. Graves, Samantha J. Moore, Erik B. Bloss, Brett D. Mensh, William L. Kath, and Nelson Spruston. Hippocampal pyramidal neurons comprise two distinct cell types that are countermodulated by metabotropic receptors. *Neuron*, 76(4):776–789, Nov 2012.

[6] Ivan Soltesz and Attila Losonczy. Ca1 pyramidal cell diversity enabling parallel information processing in the hippocampus. *Nature Neuroscience*, 21(4):484–493, 2018.

[7] Edward Y Chen, Christopher M Tan, Yan Kou, and et al. Enrichr: interactive and collaborative html5 gene list enrichment analysis tool. *BMC Bioinformatics*, 14:128, 2013.

[8] Seth Carbon, Amelia Ireland, Christopher J Mungall, Suzanna Shu, Bill Marshall, Suzanna Lewis, AmiGO Hub, and Web Presence Working Group. Amigo: online access to ontology and annotation data. *Bioinformatics*, 25(2):288–289, 2009.

[9] Robert W. Holz and Stephen K. Fisher. *Synaptic Transmission.* Lippincott-Raven, Philadelphia, 6th edition, 1999.

[10] Paul Hoffman. *pbmc3k: Raw and Processed Matrices of the PBMC 3k Dataset*, 2024. R package version 0.1.0.

[11] Satija Lab. Pbmc 3k tutorial, n.d.

[12] Dai M;Pei X;Wang XJ;. Accurate and fast cell marker gene identification with cosg. *Briefings in bioinformatics.*

[13] K. R. Karlmark, F. Tacke, and I. R. Dunay. Monocytes in health and disease - minireview. *European Journal of Microbiology and Immunology (Bp)*, 2(2):97–102, 2012.

[14] N. M. Mahaweni, T. I. Olieslagers, I. O. Rivas, et al. A comprehensive overview of fcgr3a gene variability by full-length gene sequencing including the identification of v158f polymorphism. *Scientific Reports*, 8:15983, 2018.

[15] B. Lamarthée, J. Callemeyn, Y. Van Herck, et al. Transcriptional and spatial profiling of the kidney allograft unravels a central role for fcyriii+ innate immune cells in rejection. *Nature Communications*, 14:4359, 2023.
